# Alphafold Predictions Provide Insights into the Structural Features of the Functional Oligomers of All Members of the KCTD Family

**DOI:** 10.3390/ijms232113346

**Published:** 2022-11-01

**Authors:** Luciana Esposito, Nicole Balasco, Luigi Vitagliano

**Affiliations:** 1 Institute of Biostructures and Bioimaging, CNR, Via Pietro Castellino 111, 80131 Naples, Italy; 2 Institute of Molecular Biology and Pathology, CNR c/o Department of Chemistry, University of Rome Sapienza, P.le A. Moro 5, 00185 Rome, Italy

**Keywords:** protein structure prediction, oligomeric state, protein structure–function

## Abstract

Oligomerization endows proteins with some key properties such as extra-stabilization, long-range allosteric regulation(s), and partnerships not accessible to their monomeric counterparts. How oligomerization is achieved and preserved during evolution is a subject of remarkable scientific relevance. By exploiting the abilities of the machine-learning algorithms implemented in AlphaFold (AF) in predicting protein structures, herein, we report a comprehensive analysis of the structural states of functional oligomers of all members of the KCTD protein family. Interestingly, our approach led to the identification of reliable three-dimensional models for the pentameric states of KCNRG, KCTD6, KCTD4, KCTD7, KCTD9, and KCTD14 and possibly for KCTD11 and KCTD21 that are involved in key biological processes and that were previously uncharacterized from a structural point of view. Although for most of these proteins, the CTD domains lack any sequence similarity, they share some important structural features, such as a propeller-like structure with a central cavity delimited by five exposed and regular β-strands. Moreover, the structure of the related proteins KCTD7 and KCTD14, although pentameric, appears to be characterized by a different organization of the CTD region, with the five chains forming a circle-like structure with a large cavity. Our predictions also suggest that other members of the family, such as KCTD10, KCTD13, and TNFAIP1, present a strong propensity to assume dimeric states. Although the structures of the functional oligomers reported herein represent models that require additional validations, they provide a consistent and global view of KCTD protein oligomerization.

## 1. Introduction

The occurrence of an intimate relationship between the structure and the function of proteins is the paradigmatic assumption of structural biology. Indeed, a full understanding of protein activities cannot be achieved without a profound knowledge of their structural and dynamic properties. Although fundamental advances have characterized this field in the last half a century, the experimental structural characterization of these intricate macromolecules has proven to be a difficult and lengthy process.

Fortunately, the development and the release of effective predictive methods, based on machine learning techniques, such as those implemented in the AlphaFold algorithm [1,2], which are able to generate reliable three-dimensional models of protein structures starting from their amino acid sequences, are opening new and previously unforeseen scenarios in structural biology. Particularly appealing is the perspective that, in the AlphaFold era, comprehensive approaches aimed at revealing the structural similarities/dissimilarities among all members of a protein family have become feasible at the global level.

Very recently, we applied this approach to the KCTD (proteins containing a (K)Potassium Channel Tetramerization Domain) family [3]: a class of proteins involved in an uncountable number of key physio-pathological pathways [4,5,6,7,8]. Traditionally seen as proteins involved in neurological and neurodevelopmental processes [9], more recent analyses have demonstrated their involvement in many other pathological states, including cancer, obesity, and genetic diseases [10,11,12,13,14,15,16,17]. The defining feature of these proteins is the presence in all members of the family of a BTB/POZ domain, which is commonly involved in protein (hetero)-oligomerization [18,19,20]. In the sequences of KCTD proteins, this domain is followed by other apparently unrelated and likely folded regions (C-terminal domain, CTD). While the BTB domains of these proteins have been extensively characterized, for example, by analogy with homologous domains present in other proteins, information on the CTD regions is only sporadic, as is also the case for the diversified properties of this domain in KCTDs. Over the years, the lack of structural data has negatively impacted on the understanding of the activities of these proteins at the atomic level. It is commonly believed that several KCTDs operate as substrate adaptors in protein ubiquitination by binding Cullin 3 with the BTB domains and the substrate to be labelled with the CTD [18,21,22]. However, the ability of the KCTD BTB domains to bind Cullin 3 is not a universal property within the family [23]. KCTDs, whose BTB domain is unable to recognize Cullin 3, operate in alternative partnerships, such as those established with the transcription factor AP-2α or with the GABA_B2_ receptor [8,24,25,26,27,28].

**Figure 1:**
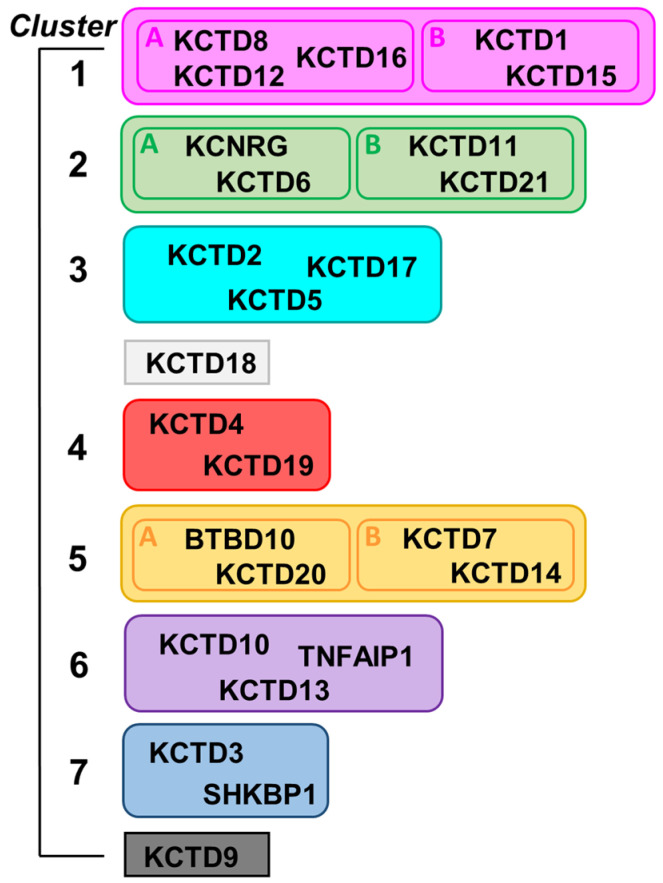
Scheme of the structure-based clustering of KCTD proteins derived from data reported in Reference [3].

By exploiting AlphaFold-predicted three-dimensional structures of single polypeptide chains of KCTDs, we recently revealed previously undetected molecular relationships among the CTD regions of the members of the family [3]. Indeed, in contrast to the previous belief, the CTD domains of most of KCTDs share some structural similarities despite the frequent absence of sequence similarity. This finding has led to the generation of a novel structure-based pseudo-phylogenetic tree, which revisited previous associations of the members of the KCTD family and grouped them in seven distinct clusters and two isolated proteins (Figure 1) [3].

A common CTD region was identified in all members of the family with the exception of KCTD3, SHKBP1, and KCTD9, which were therefore designated as noncanonical KCTDs. Although the identification of previously undetected relationships among KCTDs represented an important step in the understanding of the activities at the molecular/atomic level, it is important to note that all these proteins seem to operate as oligomers. Indeed, although the characterization of the oligomeric state of these proteins has proven to be remarkably difficult, the extrapolation of data collected mostly on the BTB domains has led to the consensus that these proteins, or at least most of them, are functionally active as pentamers. By exploiting recent functionalities in AlphaFold2 (AF) predicting tools [1,29], we report herein an exhaustive prediction of the structures of KCTD functional oligomers. In most of the cases, reliable three-dimensional structures were obtained. Extensive molecular dynamics (MD) simulations were performed to gain further insights into the reliability and the local stability of these models and into their dynamic properties. Collectively, the findings described herein shed a new and global light on the structural properties of these proteins, which play crucial roles in many biological processes.

## 2. Results

The prediction of the three-dimensional states of the functional oligomers of KCTD proteins was performed using the AlphaFold algorithm [1], as implemented in Colab [29]. Predictions were conducted by considering the clustering scheme we recently derived for the KCTD protein family (Figure 1). Predictions were conducted for all members of the family with the exception of KCTD19, whose articulated domain organization [3] makes oligomerization unlikely. Moreover, they were conducted in a step-wise manner by initially considering the frequently characterized BTB domains and, subsequently, the full-length proteins (see Section 4 for details).

### 2.1. Prediction of the Structures of the Pentamers Formed by the BTB Domains

Initial predictions were conducted by investigating the oligomerization propensities of isolated BTB domains of KCTDs as a significant amount of experimental and structural studies have been performed on this domain. Notably, BTB domains of KCTD proteins are characterized by a remarkable versatility in their oligomeric organizations [20,30,31]. Indeed, depending on the external conditions the same BTB domain may assume different oligomeric states. However, the functional relevance of this variability is yet to be assessed and it should also be considered that these findings may be due to artifacts generated from the extraction of these domains from the full-length protein contexts. In any case, distinct investigations have independently suggested that KCTD proteins mostly form pentamers in their functional states, which are characterized by a C5 symmetry [20,30,32]. In this scenario, we preliminary tested the ability of AlphaFold, as implemented in Colab [29], to predict the three-dimensional structure of possible pentamers formed by the BTB domains. As shown in Figures S1 and S2, for most of these domains, stable and reliable pentamers were predicted. This is also true for BTB domains that form other oligomers in the crystalline states, such as KCTD13^BTB^/KCTD10^BTB^ (a tetramer formed by a dimer of dimers) and SHKBP1^BTB^ (monomer) [20]. It is important to note, however, that an experimental pentameric state for the BTB of these KCTDs was detected in the presence of Cullin 3, a functional partner of these proteins [20]. Importantly, in some cases, these BTB domains have been experimentally found to be able to form open pentamers in which two adjacent subunits of the C5 assembly do not form tight contacts. Although, for some KCTDs, the opening of the close pentamer is believed to be caused by protein truncation and the consequent destabilization of the assembly, for the members of the sub-Cluster 1A (KCTD8, KCTD12, and KCTD16), the opening of the pentamer is functionally important as it favors the binding of the GABA_B2_ receptor [26,27]. Therefore, although AF predictions provide reliable structures of the pentameric states of the BTB domain of KCTD proteins, they are unable to reproduce the intrinsic ability of the BTB domains of sub-Cluster 1A to form open states, at least when the individual domains are considered.

Inspection of Figures S1 and S2 also reveals that unstable/unreliable pentameric states are predicted for some KCTDs, namely, KCTD18, an isolated member of the family, and BTBD10/KCTD20 (sub-Cluster 5A). This finding suggests that these proteins operate in nonpentameric states.

### 2.2. Prediction of the Structures of the Pentamers Formed by Full-Length KCTD Proteins

AF predictions of the three-dimensional structures of the pentamers formed by KCTDs were performed for all members of the family, with the exception of the KCTD19 protein, which presents a globular multidomain shape (see above) [3]. As typically done in AF protocols, the reliability of the predictions was assessed on the basis of the Local Distance Difference Test (LDDT) and the Predicted Aligned Error (PAE) matrices. In addition to the visual inspection of the cartoon representation of the models made using the LDDT scheme color and of the PAE matrices (Figures S3 and S4) [1,2,29], these matrices were also quantitatively elaborated to evaluate the distribution of the expected errors (see Figure S5 for an illustrative example). For each model, the percentage of distances with estimated errors lower than 10 Å was evaluated (Table S1). This analysis was conducted by separately considering distances between residues of the same domain (intra-BTB or intra-CTD) or of adjacent domains (inter-BTB or inter-CTD). Typically, 3D models were considered reliable when this percentage was higher than 65% (Table 1).

Considering the molecular and structural complexity of these proteins and their diversification, AF predictions and the resulting three-dimensional models are illustrated in the following paragraphs, separately considering the members of each cluster of the family (Figure 1).

#### 2.2.1. Cluster 1

For this cluster, in addition to the structural data existing for the BTB domains, a significant amount of experimental data are also available for the CTD domains of members of the sub-Cluster 1A, such as KCTD8 (PDB entry 6g57), KCTD12 (PDB entries 6qzl, 6m8s) [26], and KCTD16 (PDB entry 6qb7), and for full-length KCTD1 (PDB entry 6s4l).

Since in the sequences of the proteins of the sub-Cluster 1A (KCTD8, KCTD12, and KCTD16) the BTB and the CTD domains are separated by a large unstructured stretch, information on the CTD was derived by carrying out AF predictions on the isolated domain. The visual inspection of the PAE matrices (Figure S3), as characterized by the prevalence of the blue color which is characteristic of low estimated errors, indicates that, with the exception of few loop regions, the predicted structures of these pentamers are highly reliable. This indication is corroborated by the quantitative analysis of the PAE matrices for these proteins (Table S1). Low expected errors were detected, not only within individual CTD domains (intra-CTD), but also between adjacent CTD domains (inter-CTD).

In line with the crystallographic models of the CTD domain of KCTD12 (PDB entry 6qzl) and KCTD16 (PDB entry 6qb7), all these models are characterized by a propeller-like structure (Figure S6). Root mean square deviation (RMSD) values computed on the C^α^ atoms against the respective crystal structures are 0.63 Å (KCTD12, 434 atoms aligned) and 1.10 Å (KCTD16, 473 atoms aligned). The five chains of the proteins assemble to generate a central cavity that is delimited by the terminal β-strands of the β-sheet, which constitute the blades of the propeller. This particular structural arrangement generates cavities that are characterized by edges containing fully exposed H-bond donors and acceptors. This is a rather peculiar assembly as regular and exposed β-strands are considered particularly sticky and frequently involved in uncontrollable intermolecular interactions [33]. Particularly intriguing is an anomalous structure of the KCTD8 CTD domain reported in the PDB (PDB entry 6g57) in which two subunits of the proteins strongly interact through two of these β-strands, thus confirming their sticky nature (Figure S7).

Attempts to predict the structure of a construct with covalently connected BTB and CTD were conducted for KCTD16, the member of the family with the shortest disordered link between the two domains. Although these analyses did not provide reliable indications of the possible juxtaposition between the two domains, the pentameric structure of the BTB domain adopts an open state in these conditions (the full-length context), which is very close to the functional state crystallographically characterized (PDB entry 6ocr) with a RMSD value computed on the C^α^ atoms of 1.25 Å (425 atoms aligned) (Figure S8) [27]. This finding is suggestive of minimal energetic differences between the open and the close pentameric states of the BTB domains of these proteins, which is in line with previous MD analyses [31].

Structural predictions conducted on the members of the sub-Cluster 1B (KCTD1 and KCTD15) also led to reliable models characterized by reliable interactions both at the level of individual domains (intra-BTB and intra-CTD) and of adjacent domains (inter-BTB and inter-CTD). The predicted models of the two proteins are similar to the crystallographic structure of the full-length KCTD1 reported in the PDB (PDB entry 6s4l). Indeed, the RMSD values computed on the C^α^ atoms are 0.91 Å (KCTD1, 936 atoms aligned) and 1.46 Å (KCTD15, 940 atoms aligned).

**Figure 2:**
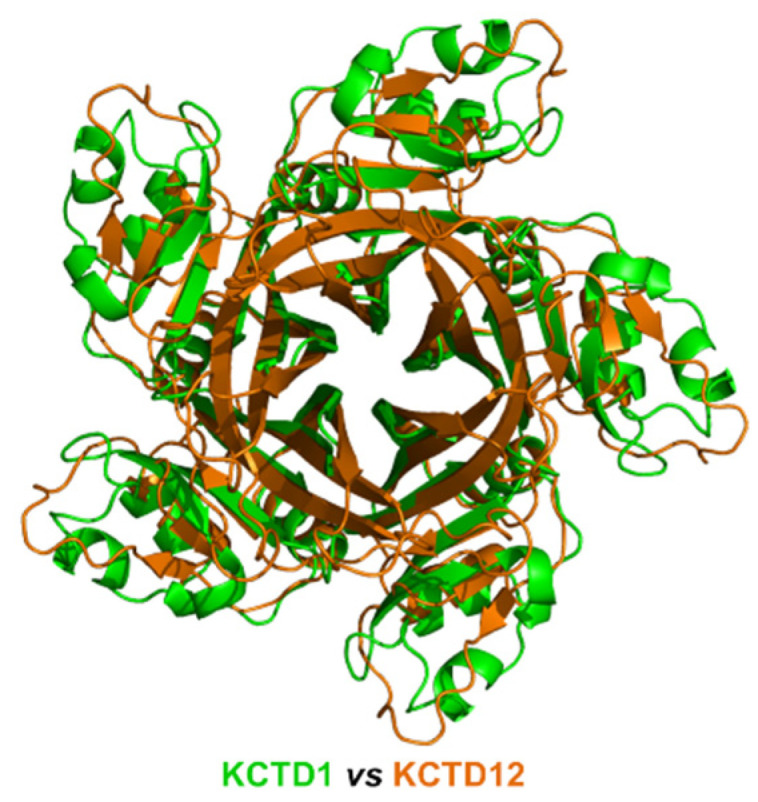
Structural superimposition of the CTD domains of KCTD1 (green) and KCTD12 (orange) obtained by combining US-align and PyMOL (see Section 4). The RMSD value computed on the C^α^ atoms (325 atoms aligned) is 2.48 Å.

Despite the limited sequence identity (<20%), which was not detected by BLAST searches but revealed by DALI structural alignments, detected between the CTD domains of the proteins of sub-Clusters 1A and 1B, their overall pentameric organization is remarkably similar (Figure 2). Indeed, the CTD domain of KCTD1/KCTD15 also presents a propeller-like fold with a central cavity delimited by five regular and exposed β-strands. However, in contrast to what was observed for the proteins of sub-Cluster 1A, the pentamers of KCTD1/KCTD15 are characterized by an intimate connection between the BTB and the CTD domains.

#### 2.2.2. Cluster 2

For members of Cluster 2, no experimental structural data are available for the CTD domain. Moreover, although the sequences of the CTD domains of KCTD11 and KCTD21 share a significant degree of sequence identity (31.6%), the sequences of the CTD domains of both KCTD6 and KCNRG do not share detectable sequence identities with each other nor with the other members of the family. They were included in Cluster 2 together with KCTD11/KCTD21 for some structural similarities that emerged from the AF models of their single polypeptide chains [3].

**Figure 3:**
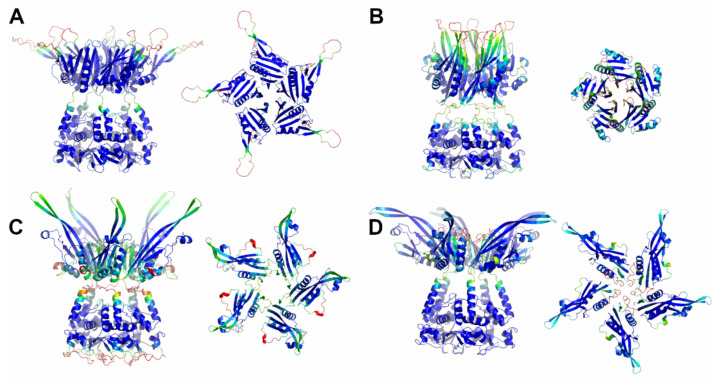
Schematic representation of AF full-length structures of members of Cluster 2. (**A**) KCNRG; (**B**) KCTD6; (**C**) KCTD11; (**D**) KCTD21. For each model, two views (on the left, the full-length pentameric assembly; on the right, the CTD domains only) are shown. Structural models are colored following AF per-residue confidence metric (pLDDT).

Here, a reliable KCNRG pentamer was derived through AF predictions (Figure S3, Table 1 and Table S1). The analysis of the PAE matrices exhibited rather low estimated errors for the intra- and interdomain contacts of both BTB and CTD pentamers. The analysis of the three-dimensional structure of these pentamers indicates that the CTD domain adopts a structure that has some important analogies with the structures of the pentamers of KCTDs of Cluster 1, despite the absence of sequence similarities. Notably, the pentamer forms a propeller-like assembly whose central cavity is surrounded by exposed β-strands (Figure 3). A similar situation was observed for KCTD6 (Figure 3), thus indicating remarkable structural analogies between the members of the sub-Cluster 2A.

The reliability of the pentamers for KCTD11/KCTD21 (sub-Cluster 2B) is somehow lower compared to the other members of the cluster (Table S1). The inspection of the structures of these two pentamers again highlights the occurrence of a propeller-like fold endowed with a central cavity. However, the cavity is only delimited by β-strands for KCTD11, whereas it is surrounded by five symmetrical irregularly structured regions in KCTD21 (Figure 3).

**Figure 4:**
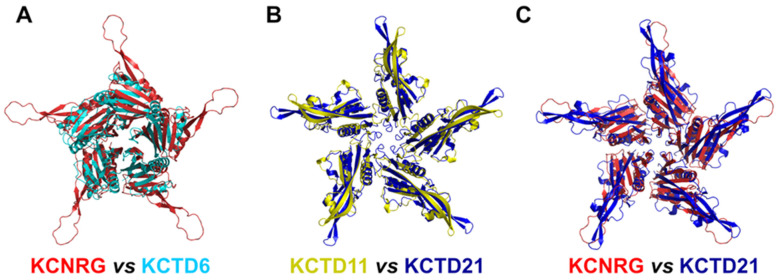
Comparison of the three-dimensional structures of the CTD domains of members of Cluster 2. (**A**) KCNRG (red) and KCTD6 (cyan), RMSD of 3.99 Å, 225 C^α^ atoms aligned; (**B**) KCTD11 (yellow) and KCTD21 (blue), RMSD of 2.36 Å, 579 C^α^ atoms aligned; (**C**) KCNRG (red) and KCTD21 (blue), RMSD of 7.62 Å, 370 C^α^ atoms aligned. The structural superimpositions were obtained by combining US-align and PyMOL (see Section 4).

A global and quantitative comparison of the CTD-predicted structures of the members of Cluster 2 highlights their similar structural organization (Figure 4). Indeed, with the exception of a few external regions, not only do the single chains of these domains exhibit a similar fold but also the pentamers are assembled in an analogous fashion.

Since no experimental information was available for the CTD domains and the full-length proteins, these predicted AF structures were subjected to MD analyses (see below).

#### 2.2.3. Cluster 3

Reliable models have been obtained for the three members of this cluster (KCTD2, KCTD5, and KCTD17) (Figures S3 and S9). For full-length KCTD5, two distinct experimental structures were determined, in high (PDB entry 3drx) and low (PDB entry 3dry) salt conditions, which present a different orientation of the CTD compared to the BTB domain [34]. The possibility of the two domains exhibiting a rotation-like motion was corroborated by MD simulations [35]. The predicted structure of full-length KCTD5, although intermediate between these two states, is closer to the high salt crystallographic form with a RMSD value of 1.44 Å (753 atoms aligned) vs. 4.06 Å (790 atoms aligned).

The predicted models of KCTD2 and KCTD17 (Figure S9) present three-dimensional structures that are very similar to that displayed by KCTD5. Collectively, all these pentamers are characterized by the presence of a central cavity that is surrounded by a bunch of regular and exposed β-strands.

#### 2.2.4. Cluster 4 and KCTD18

As anticipated above, analyses of the KCTD19 oligomerization were not performed; therefore, from the Cluster 4 proteins, only the pentameric state of KCTD4 was predicted. As reported in Figure S3 and Table S1, the inspection of the PAE matrices indicates that rather low errors were estimated for the intra-BTB and CTD interactions. Similarly, low errors were expected for the inter-BTB interactions. The expected errors of the inter-CTD interactions, although significantly larger than those detected for inter-BTB, indicate that this model still retains an acceptable level of accuracy (see the MD analysis below).

**Figure 5:**
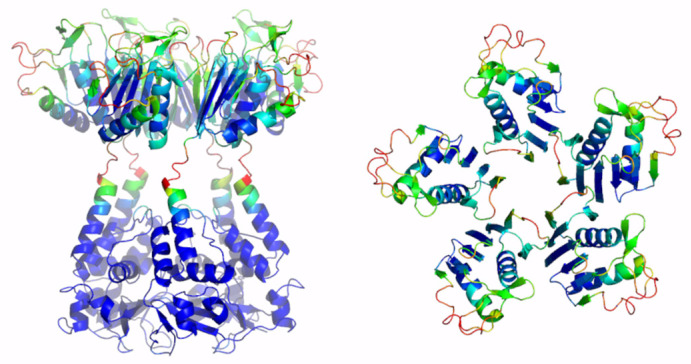
Schematic representation of AF full-length KCTD4. Two views are shown (on the left, the full-length pentameric assembly; on the right, the CTD domains only). The structural model is colored following AF per-residue confidence metric (pLDDT).

Again, the three-dimensional structure of the KCTD4 pentamer is characterized by a propeller-like fold with a central cavity surrounded by regular and exposed β-strands (Figure 5).

Attempts to predict the structure of a putative KCTD18 pentamer resulted in a highly unreliable model characterized by large expected errors between its different chains (Figure S4). The only reliable portion of this model is the structure of the individual chains of the BTB and, partially, of the CTD domains.

#### 2.2.5. Cluster 5

The predictions carried out for the putative pentameric states of KCTDs of Cluster 5 provide quite different results depending on the protein distribution in the two subclusters. Indeed, attempts to reconstruct oligomers for the members of sub-Cluster 5A (BTBD10 and KCTD20) did not lead to the identification of stable and reliable pentamers (Figure S4). However, a deeper inspection of the PAE matrices suggests that the CTD domains of these proteins have a significant tendency to interact with an adjacent CTD mate. In both proteins, recurrent interactions between the BTB and the CTD domains of adjacent chains generate a sort of open multimer whose reliability needs to be experimentally assessed in future studies.

**Figure 6:**
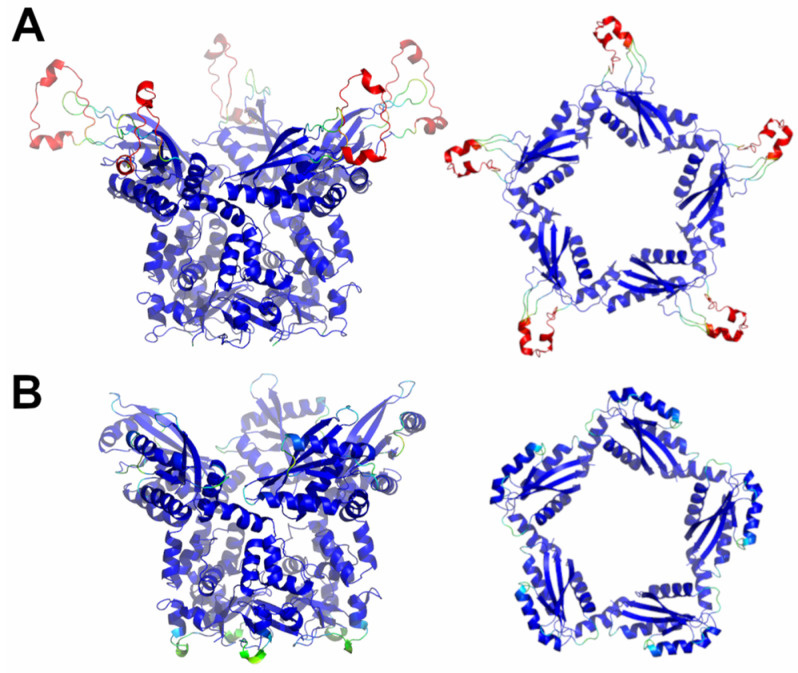
Schematic representation of AF full-length structures of members of sub-Cluster 5B. (**A**) KCTD7; (**B**) KCTD14. For each model, two views (on the left, the full-length pentameric assembly; on the right, the CTD domains only) are shown. Structural models are colored following AF per-residue confidence metric (pLDDT).

A completely different scenario emerges from the prediction analyses conducted on putative KCTD7 and KCTD14 pentamers (sub-Cluster 5B). As shown in Figure S3, these proteins form assemblies characterized by rather reliable inter- and intra-BTB and CTD contacts. The analysis of the three-dimensional structures of these pentamers highlights significant differences with the pentameric assemblies formed by the KCTD proteins, which were described in the previous paragraphs. Indeed, despite the canonical pentameric organization of the BTB domains of these proteins, the five CTD units self-assemble by forming a large central cavity (Figure 6), which is completely different from those detected in KCTD proteins of Clusters 1–4.

Globally, both KCTD7 and KCTD14 assume structures that resemble a Greek krater with the CTD pentamer forming the top. The global features of the fold of the CTDs and their associations in the pentamer are well shared by these two proteins (Figure S10). Since there is no experimental evidence about the formation of these pentameric structures, they were further evaluated using MD simulations (see below).

#### 2.2.6. Cluster 6

AF predictions carried out to assess the putative pentameric assemblies of the proteins of Cluster 6 (KCTD10, KCTD13, TNFIAP1) were unsuccessful. Indeed, irregular and unreliable pentameric states were predicted for these proteins (Figure S4). However, the presence of quadrants with low estimated errors in the top-left and in the low-right regions of the matrices is suggestive of the propensity of these proteins to form stable dimers. This possibility is explored below.

#### 2.2.7. Cluster 7 and KCTD9

This group of proteins was denoted as noncanonical KCTDs since their sequences do not present folded regions that resemble the CTDs that have been detected, along with the BTB domains, in all other members of the family.

The C-terminal region of the two members of Cluster 7 (KCTD3 and SHKBP1) corresponds to a large (approximately 350 residues) and well-folded domain that assumes an eight-bladed β-propeller structure. Considering the size and the defined structure of this domain, it is unlikely that it undergoes oligomerization. Since the BTB domain of these proteins may form stable pentamers, as experimentally demonstrated [20] and confirmed by AF predictions herein, by modelling, we verified that such a large C-terminal domain is compatible with a global pentameric structure of KCTD3 and SHKBP1.

**Figure 7:**
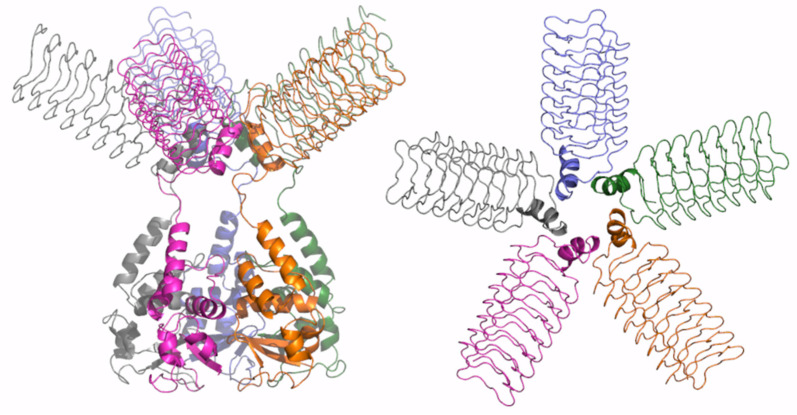
Schematic representation of AF full-length structure of KCTD9. The structural model was obtained by combining the AF predictions of the protein with a reduced CTD domain (residues 89–282) and of the whole CTD domain (residues 199–380). Two views are shown (on the left, the full-length pentameric assembly; on the right, the CTD domains only); the model is colored by chain.

In addition to the BTB domain, we previously predicted that KCTD9 presents two other folded domains: (a) a ubiquitin-like at the N-terminus and (b) a pentapeptide repeat domain of the β-solenoid class at the C-terminus. Since the overall size of the protein prevents predictions of the full-length form, to gain insights into its oligomerization propensity, we carried out prediction trials by dissecting KCTD9 in different fragments. Since, as shown above, the KCTD9 BTB domain presents a remarkable propensity to form a pentamer, we initially analyzed the ability of the assembly formed by the ubiquitin-like and the BTB domain to form a pentamer. This first trial, while confirming the tendency of the BTB to form stable pentamers, showed that the ubiquitin-like domain was unable to form a reliable and symmetric pentamer (Figure S11). On the other hand, the construct formed by the BTB and a significant portion of the pentapeptide repeat domain, which was initially truncated to reduce the required computational resources, generated a very stable and reliable pentamer (Figure S12). A full-length pentamer of this region of the protein was generated by molecular modelling (Figure 7).

### 2.3. Putative Dimeric States Predicted for Full-Length Cluster 6 Members

As outlined in the previous paragraphs, most of the KCTD proteins are predicted to form stable pentamers. In some specific cases, as for the members of Cluster 6 (KCTD10, KCTD13, TNFIAP1), AF predictions have raised the possibility that these proteins could self-assemble in dimeric states. Ad hoc AF predictions carried out to either corroborate or confute this possibility indeed show that these KCTD proteins likely operate as dimers.

**Figure 8:**
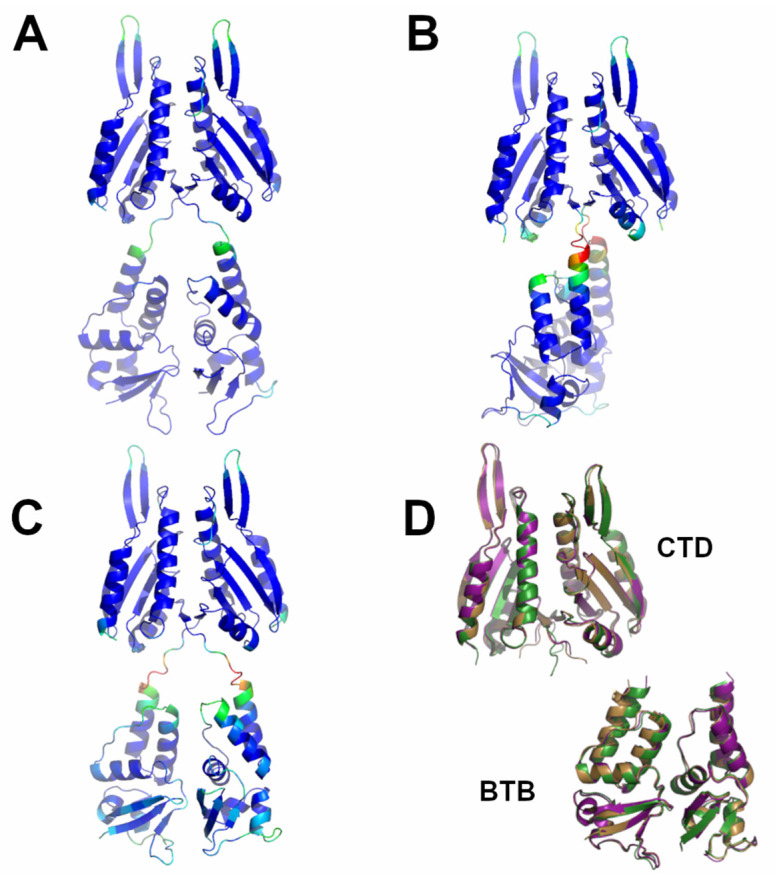
Schematic representation of AF full-length structures of members of Cluster 6. (**A**) KCTD10; (**B**) KCTD13; (**C**) TNFAIP1; (**D**) superimposed CTD and BTB domains (KCTD10 in purple; KCTD13 in dark green; TNFAIP1 in sand). Structural models in panels A-C are colored following AF per-residue confidence metric (pLDDT).

As shown in Figure S13, the prediction matrices of Cluster 6 dimers are suggestive of reliable contacts, not only for intra-BTB and CTD, but also for inter-CTD. This finding suggests that the association between the C-terminal domains constitutes the driving force of dimer formation for these two proteins. This observation is confirmed by the inspection of the predicted three-dimensional structures of these dimers (Figure 8). The interface area buried upon dimer formation is remarkably ~1700 Å^2^ per subunit. A key role in dimer formation is the association of two external β-strand regions of the two subunits.

### 2.4. Molecular Dynamics Simulation on Newly Predicted KCTD Oligomerization States

In order to validate the new structural states generated by the AF predictions and to check their local stability, we performed fully atomistic MD simulations in explicit solvent (timescale 200 ns) for all assemblies formed by proteins whose CTD domains do not share significant sequence similarities with the KCTDs that have been experimentally characterized. In particular, simulations were performed on the pentameric states of KCNRG (Figure S14), KCTD6 (Figure S15), KCTD11 (Figure S16), KCTD21 (Figure S17), KCTD4 (Figure S18), KCTD7 (Figure S19), and KCTD14 (Figure S20), and on the dimeric state of KCTD13 (Figure S21). Considering the elevated similarities of the members of Cluster 6, simulations were not performed on KCTD10 and TNFIAP1. Moreover, the large size of the pentamer formed by KCTD9 also prevented the possibility of satisfactorily sampling this assembly.

A global analysis of the simulations carried out on the pentameric assemblies, in terms of (a) RMSD values computed against the starting AF model and (b) the evolution of the secondary structure elements, clearly indicates that, for most of them, the overall integrity of the pentamers formed by the BTB and CTD domains is well retained. Indeed, considered individually, the pentamers formed by these domains present low (<3 Å) or limited (<6 Å) RMSD values when compared to the starting model. It is also important to note that loop regions significantly contribute to the increase in these values. The only significant exceptions to this trend are observed for KCTD11 and, in part, for KCTD21, which also present the lowest PAE score (Table S1) among this ensemble of structures. Collectively, these findings provide a strong support to the AF-predicted models of CTD oligomers with unknown structures. Further analyses are required to assess the reliability of KCTD11 and KCTD21 models.

The analysis of the RMSD values computed for the entire pentameric assemblies (Figures S14A–S20A) of these proteins highlights that for KCTD14, they are very low and comparable to those detected for the individual BTB and CTD domains. This clearly indicates that the overall structure of this protein is remarkably rigid and that the interdomain interactions were correctly predicted by AF. For the other proteins (KCNRG, KCTD6, KCTD4, and KCTD7) the global RMSD values, although larger compared to KCTD14, are not dramatically different from those observed for the individual domains. Moreover, the comparison of the trajectory models that are the closest to the average structure shows that a motion corresponding to a relative rotation of the CTD and the BTB domains occurs. This closely resembles the interdomain motions that were previously revealed, both experimentally and computationally, for KCTD5 [34,35].

The analysis of the MD simulation performed on the KCTD13 dimer clearly indicates that the dimer formed by the two domains is very stable (Figure S21). The secondary structure is also well preserved. However, the overall RMSD values of the trajectory structures compared to the starting one are rather high. The comparison between the trajectory structure closest to the average and the starting model shows that a bending motion of the global structure of the dimer occurs.

In conclusion, these analyses generally confirm the reliability of the AF models and also provide indications on their rigidity and preferred motions.

## 3. Discussion

Oligomerization endows proteins with some fundamental properties, such as extra-stabilization, long-range allosteric regulation, and specific partnerships, which are not accessible to the monomers. Not surprisingly, the percentage of proteins that operate as oligomers greatly increases, from primitive and simple organisms as prokaryotes to high eukaryotes. How oligomerization is achieved and preserved during evolution is a subject of remarkable scientific relevance [36].

**Figure 9:**
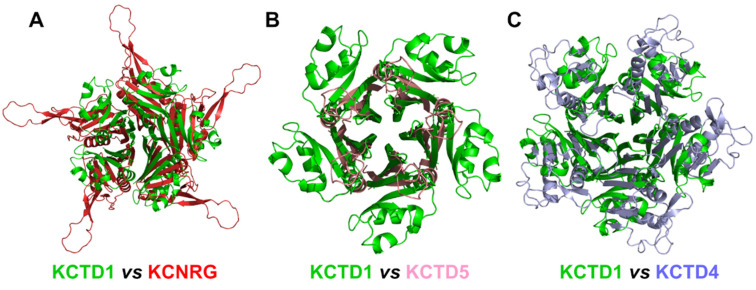
Structural alignment of the CTDs of KCTD1 (green) with (**A**) KCNRG (red), RMSD of 25.07 Å, 417 C^α^ atoms aligned; (**B**) KCTD5 (pink), RMSD of 8.14 Å, 188 C^α^ atoms aligned; (**C**) KCTD4 (light blue), RMSD of 12.73 Å, 314 C^α^ atoms aligned. The structural superimpositions were obtained by combining US-align and PyMOL (see Section 4).

Herein, by exploiting the prediction ability of the AlphaFold algorithm, we explored the possible three-dimensional organization of the functional oligomers formed by KCTD proteins. Although these proteins have been identified as key players in different physio-pathological contexts, the structural characterization of their functional states has been hitherto quite limited. Even the characterization of the oligomeric state of their functional states has been sporadic and frequently limited to domain fragments [20,30,32]. Nevertheless, it is commonly believed that most of these proteins form functional oligomers. Therefore, based on these observations, pentameric structures for 24 out of the 25 KCTD proteins were predicted herein. Interestingly, in most cases, reliable models were generated. Indeed, in addition to producing pentameric structures of the BTB domains that were in good agreement with the experimental ones, AF was able to reproduce all of the structural features of the few KCTD proteins whose CTD region had been crystallographically characterized [26,34, KCTD1–PDB entry 6s4l]. Importantly, herein, we report reliable three-dimensional models for pentameric KCTDs, such as KCNRG, KCTD6, KCTD4, KCTD7, KCTD14, and KCTD19 and possibly for KCTD11 and KCTD21, which are involved in key biological processes [9,10] and were previously uncharacterized from a structural point of view. Interestingly, the CTD domains of KCTD proteins belonging to Clusters 1, 2, 3, and 4, despite the lack of any sequence similarity, share some important structural features, such as a propeller-like structure with a central cavity delimited by five exposed and regular β-strands (see Figure 9 for some illustrative comparisons). The preservation of this motif is particularly intriguing when considering that the CTD domains of the proteins in Cluster 1 and 3 also differ dramatically in size (~100–120 residues vs. ~60 residues).

The structure of the related proteins and previously structurally uncharacterized KCTD7 and KCTD14 (sub-Cluster 5B), although pentameric, appears to be characterized by a different organization of the CTD region. Indeed, the five chains of the terminal domain of these proteins, whose deficiency, in particular that of KCTD7, has been associated with a progressive neurodegenerative disorder and epilepsy [37,38,39], associate forming a circle-like structure with a larger cavity compared to that found in the pentamers of the KCTDs of Clusters 1–4. Our predictions highlight the peculiar oligomerization properties of the members of Cluster 6 (KCTD10, KCTD13, and TNFAIP1), which are involved in important neurological pathologies including autism [6,7,40,41]. In line with some experimental indications [20], the BTB domains of these proteins are able form stable pentamers. However, when the full-length proteins are considered, these KCTDs seem to prefer a dimeric state that is strongly stabilized by interactions formed by the CTD regions. Interestingly, the dimeric association of the BTB domains in these structures closely resembles the one detected in the crystallographic structures of the isolated domains (PDB entries 5fta for KCTD10 and 4uij for KCTD13). The RMSD values computed on the C^α^ atoms of the BTB against these crystallographic structures are 0.56 (KCTD10, 188 atoms aligned) and 0.51 (KCTD13, 191 atoms aligned).

A survey of the PDB for similar structural assemblies highlights a close similarity between the CTD pentamer of the members of Cluster 1 and that formed by the GTP cyclohydrolase I feedback regulatory protein [42,43]. No other pentameric structure of the PDB resembles the ones predicted herein. Intriguingly, the individual CTD subunits have strong similarities with the thioredoxin folds, in particular with those of the resurrected ancestral thioredoxin such as that of the Last Eukaryotic Common Ancestor [44].

Although the structures of the functional oligomers reported herein represent models that require additional validation, they provide a consistent and global view of the oligomerization of the KCTD proteins. Indeed, starting from the members of Cluster 1, we observe variations on a common theme when the members of Clusters 2, 3, and 4 are comparatively analyzed. When the divergence in the structure-based pseudo-phylogenetic tree reported in Figure 1 increases, as in Cluster 5, either the full-length pentamers are no longer stable (sub-Cluster 5A) or they display a different organization (sub-Cluster 5B). The propensity to form full-length pentamers is completely lost for members of Cluster 6. The noncanonical KCTDs of Cluster 7 may form pentameric assemblies, likely dictated by the propensity of their BTB domains to assume these oligomeric states. In KCTD9, the pentameric state of the BTB domain likely extends the large pentapeptide repeat domain of the β-solenoid class of the C-terminal regions.

Finally, the present study also indicates the feasibility of studies aimed at exploring the structural basis of KCTD partnerships and their ability to form hetero-oligomers that are able to modulate the activity of these proteins [45,46].

## 4. Materials and Methods

### 4.1. AlphaFold Predictions

The definition of the sequence stretches corresponding to the BTB and the CTD regions of the KCTD proteins are taken from the study of Esposito et al. [3]. Their clustering is reported in Figure 1.

Three-dimensional structures of KCTD oligomers were predicted using the AlphaFold v2.0 algorithm as implemented on the Colab server (https://colab.research.google.com/github/sokrypton/ColabFold/blob/main/AlphaFold2.ipynb) [29] we accessed on 1 May 2022.

Predictions of KCTD oligomers were initially carried out on the BTB domains, for which substantial literature data are available, and were then extended to include the CTD domains (herein, the BTB+hinge+CTD are referred as the full-length protein). To save computational resources, predictions were performed by initially assuming a pentameric state for all members of the family.

Predictions were performed without considering any homologous experimental template (template_mode: none) and with three as the number of recycles. The best predicted model (rank 1) out of the five computed by AF is considered throughout the present work.

The reliability of the AF predictions was assessed by deeply analyzing the Local Distance Difference Test (LDDT) score and the Predicted Aligned Error (PAE) matrices reported for each structure of the AF database.

### 4.2. Molecular Dynamics Simulations

#### 4.2.1. Systems

Predicted KCTD oligomers were used as starting models for fully atomistic Molecular Dynamics (MD) simulations carried out in explicit solvent. Simulations were performed on the pentameric states of KCTD4, KCTD6, KCTD7, KCTD11, KCTD14, and KCNRG and on the dimeric state of KCTD13.

#### 4.2.2. Parameters

The GROMACS software (v2020.3) was used to carry out fully atomistic MD simulations on KCTD proteins using the Amber99sb all-atom force field [47]. The protein models were solvated with water molecules of the TIP3P model in triclinic boxes and neutralized with counterions (sodium or chloride). The Particle Mesh Ewald (PME) method (0.16 nm grid spacing) was used to treat the electrostatic interactions [48], whereas a cut-off of 10 Å was applied for Lennard–Jones interactions. The LINCS algorithm was used to constrain bond lengths [49]. Systems were energy minimized using steepest descent for 50,000 steps. Then, they were equilibrated in two steps. The temperature was raised to 300 K in 500 ps (NVT ensemble) and then the pressure was equilibrated at 1 atm in 500 ps (NpT ensemble). The Velocity Rescaling and Parrinello−Rahman algorithms were applied for the control of temperature and pressure, respectively. MD production runs (timescale of 200 ns) were performed at constant pressure (1 atm) and temperature (300 K) using an integration time step of 2 fs. Structural analyses of MD trajectories were performed using GROMACS tools and the Visual Molecular Dynamics (VMD) program [50].

Figures of structural models were generated using the PyMOL molecular visualization system [51]. Structural alignments of models were carried out by the align routine of PyMOL using automatic cycles of refinement to reject structural outliers resulting from the fit. In difficult cases of oligomer superimposition, the US-align server [52] was used to identify the best aligned regions in the monomers, which were then used in the align routine of PyMOL to superimpose the full pentamers. Plots were generated using Xmgrace v50125 (https://plasma-gate.weizmann.ac.il/Grace/, accessed on 1 May 2022) and the OriginPro 2020 software [53].

## Figures and Tables

**Table 1:** Qualitative assessment of the predictions. For each prediction in the left column, the stability of the oligomeric assembly (S = stable; U = unstable as a pentamer) is reported; the right column shows the reliability of the prediction (R = reliable; PR = partially reliable) based on a visual inspection of the PAE matrices. Proteins predicted to form stable dimers (SD) are also listed.

Protein	BTB		Full-Length ^1^	
KCTD8	S	R	S	R
KCTD12	S	R	S	R
KCTD16	S	R	S	R
KCTD1	S	R	S	R
KCTD15	S	R	S	R
KCTD6	S	R	S	R
KCNRG	S	PR	S	R
KCTD11	S	R	S	PR
KCTD21	S	R	S	PR
KCTD2	S	R	S	PR
KCTD5	S	R	S	PR
KCTD17	S	R	S	R
KCTD18	U	-	U	-
KCTD4	S	PR	S	PR
BTBD10	U	-	U	-
KCTD20	U	-	U	-
KCTD7	S	R	S	PR
KCTD14	S	R	S	R
KCTD10 ^2^	S	R	U/SD	-/PR
KCTD13 ^2^	S	R	U/SD	-/PR
TNFAIP1 ^2^	S	R	U/SD	-/PR
KCTD3 ^3^	S	R	-	-
SHKBP1 ^3^	S	R	-	-
KCTD9 ^4^	S	R	S	R

^1^ For KCTD8, KCTD12, and KCTD16, due to the long unstructured segment between domains, predictions of the full-length proteins were not performed. Data in the column refer to the prediction of pentameric CTD assemblies. ^2^ The full-length prediction results in an unstable pentamer but in a stable dimer. ^3^ For the large CTD domain of KCTD3 and SHKBP1, predicted by AF as a self-consistent monomer, we did not carry out CTD pentameric assembly predictions. ^4^ The CTD domain adopts a pentapeptide repeat structure (β-solenoid class) with a typical superhelical arrangement. For the full-length prediction, we used a reduced sequence of the CTD (residues 199–282 instead of 199–380) by considering approximately half of the coils of the right-handed quadrilateral β helix.

## Data Availability

Not applicable.

## Supplementary Materials

The following supporting information can be downloaded at: https://www.mdpi.com/article/10.3390/ijms232113346/s1, Table S1: Quantification of the reliability of the predictions through the analyses of the matrices; Figure S1: AF structures of the pentameric BTB of KCTD; Figure S2: PAE matrices of the BTB domains of KCTD proteins in the pentameric state; Figure S3: PAE matrices of all reliable pentamers of full-length KCTDs; Figure S4: PAE matrices and unreliable structures of pentameric assemblies of full-length KCTDs; Figure S5: Quantification of the reliability of the predictions through the analyses of the matrices: KCTD6 as example; Figure S6: Schematic representation of AF structures of members of Cluster 1; Figure S7: KCTD8 protein; Figure S8: KCTD16 protein; Figure S9: Schematic representation of AF structures of members of Cluster 3; Figure S10: Structural superimposition of the CTDs of members of sub-Cluster 5B; Figure S11: AF model of KCTD9 sequence including the N-terminal ubiquitin-like domain and the BTB domain; Figure S12: Schematic representation of the AF model of full-length KCTD9 with a reduced CTD; Figure S13: PAE matrices of Cluster 6 dimers; Figure S14: Structure stability of the pentameric AF model of KCNRG in the MD simulation; Figure S15: Structure stability of the pentameric AF model of KCTD6 in the MD simulation; Figure S16: Structure stability of the pentameric AF model of KCTD11 in the MD simulation; Figure S17: Structure stability of the pentameric AF model of KCTD21 in the MD simulation; Figure S18: Structure stability of the pentameric AF model of KCTD4 in the MD simulation; Figure S19: Structure stability of the pentameric AF model of KCTD7 in the MD simulation; Figure S20: Structure stability of the pentameric AF model of KCTD14 in the MD simulation; Figure S21: Structure stability of the dimeric AF model of KCTD13 in the MD simulation.
