# Supplementary material for: Alphafold Predictions Provide Insights into the Structural Features of the Functional Oligomers of All Members of the KCTD Family
Source: Int J Mol Sci. 2022 Nov 1;23(21):13346. doi: 10.3390/ijms232113346 (PMC9658877; doi:10.3390/ijms232113346)
Supplement: Supplementary file 1 [file ijms-23-13346-s001.zip › ijms-1995322-supplementary.pdf]

## Supplementary Material

**Table S1.** Quantification of the reliability of the predictions through the analyses of the matrices. The percentage of predicted aligned errors (PAE) below a defined threshold for residues pairs belonging to BTB and/or CTD domains is reported (see also Figure S5 for further details). The predictions refer to full length pentamers or dimers as indicated.

| **Protein** | **% distances with PAE < 10 Å** | | | | **with PAE < 15 Å*** |
|---|---|---|---|---|---|
| | **Intra BTB** | **Intra CTD** | **Inter BTB (AB)** | **Inter CTD (AB)** | **Inter CTD (AB)** |
| *Pentamers* | | | | | |
| **KCTD8** | | 76.1 | | 65.8 | |
| **KCTD12** | | 75.4 | | 68.5 | |
| **KCTD16** | | 79.0 | | 73.8 | |
| **KCTD1** | 98.6 | 90.4 | 97.0 | 81.5 | |
| **KCTD15** | 98.0 | 87.6 | 95.9 | 79.7 | |
| **KCTD6** | 96.3 | 81.7 | 92.0 | 71.1 | |
| **KCNRG** | 97.9 | 80.3 | 95.9 | 71.8 | |
| **KCTD11** | 81.1 | 82.6 | 75.5 | 36.2 | 69.7 |
| **KCTD21** | 98.1 | 82.5 | 96.8 | 60.0 | 80.0 |
| **KCTD2** | 97.8 | 76.7 | 94.7 | 59.2 | 76.9 |
| **KCTD5** | 91.2 | 76.7 | 88.6 | 62.4 | 77.3 |
| **KCTD17** | 94.2 | 81.0 | 90.8 | 72.2 | |
| **KCTD4** | 98.7 | 76.3 | 96.7 | 54.4 | 70.3 |
| **KCTD7** | 99.1 | 71.0 | 97.9 | 62.4** | 73.3 |
| **KCTD14** | 96.5 | 94.9 | 93.4 | 81.4 | |
| **KCTD9*** ** | 95.2 | 99.3 | 92.6 | 98.8 | |
| *Dimers* | | | | | |
| **KCTD10** | 98.9 | 95.3 | 51.3 | 91.3 | |
| **KCTD13** | 97.5 | 95.8 | 8.2 | 92.3 | |
| **TNFAIP1** | 93.1 | 96.2 | 0.0 | 92.0 | |

* For values less than 65% we have also calculated the percentage of distances using a PAE threshold of 15 Å. ** This values increases to 86.1% just by deleting the disordered region (residues 148–173). ***The prediction has been carried out on a reduced sequence (see the main text for details).

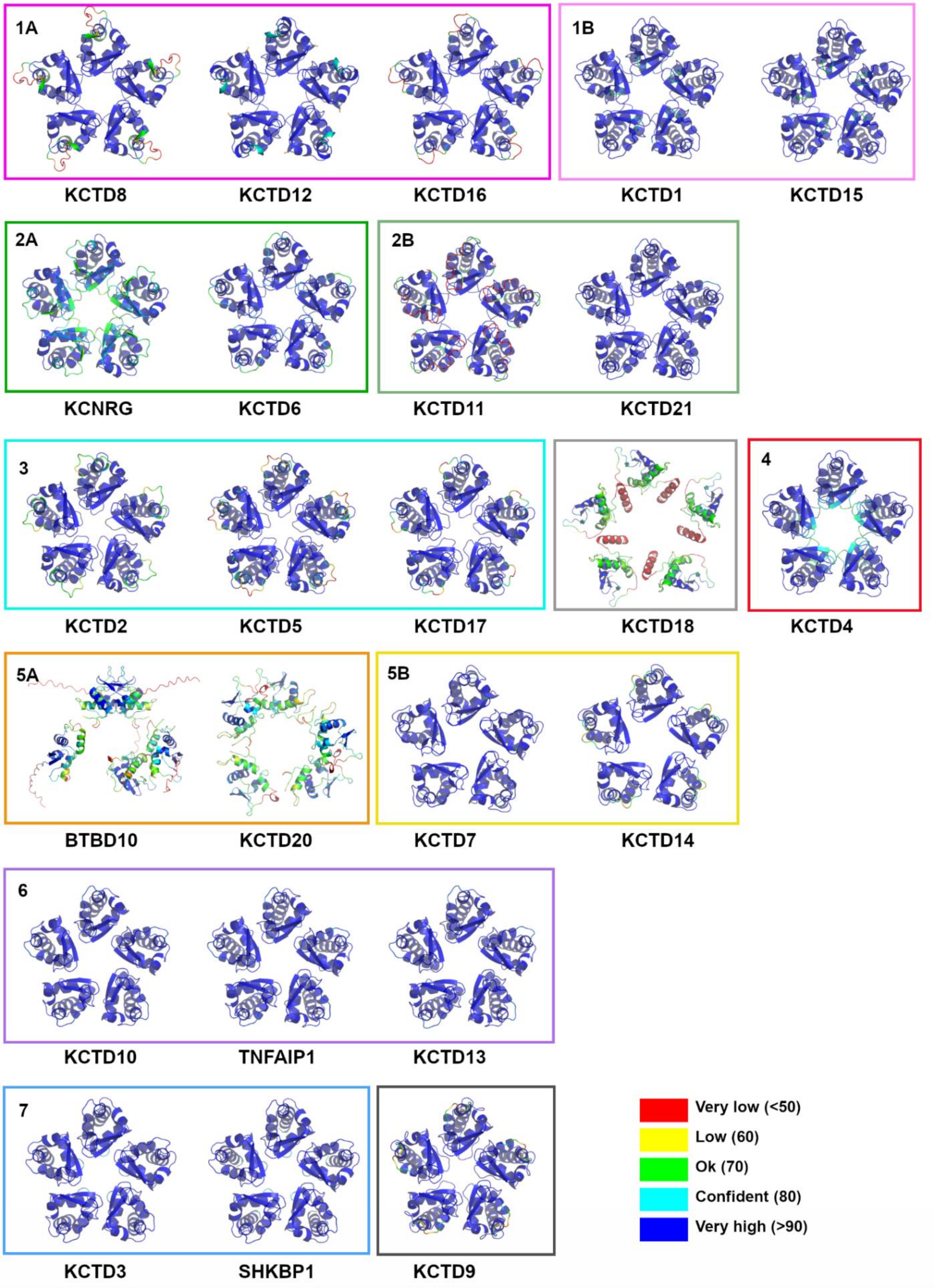


**Figure S1.** Schematic representation of AF structures of the pentameric BTB domains of KCTD proteins grouped by Cluster (see Figure 1 of the main text). Structural models are colored following AF per-residue confidence metric (pLDDT).

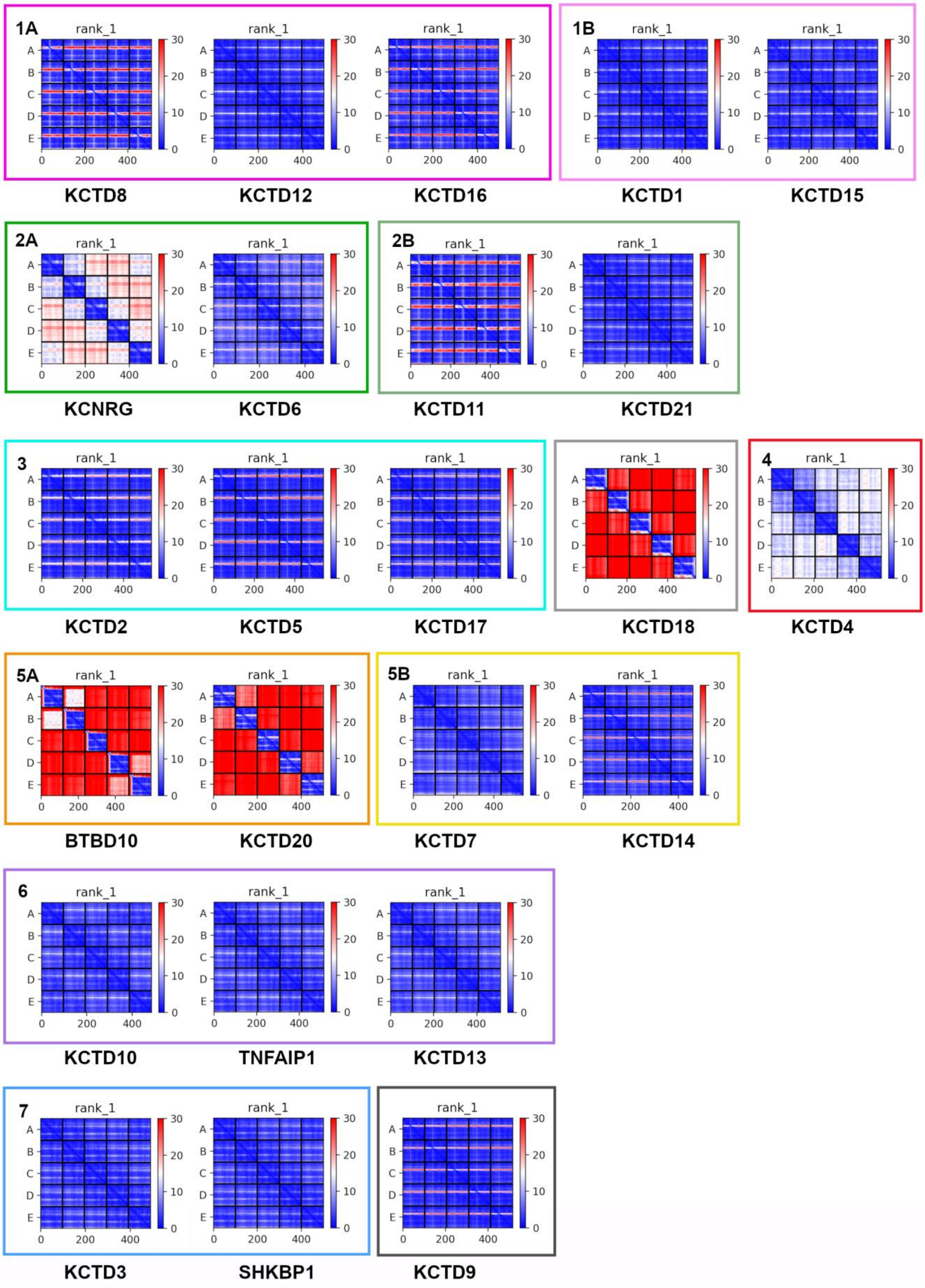


**Figure S2.** PAE matrices of the BTB domains of KCTD proteins in the pentameric state. Only the best AF prediction (rank 1) is shown.

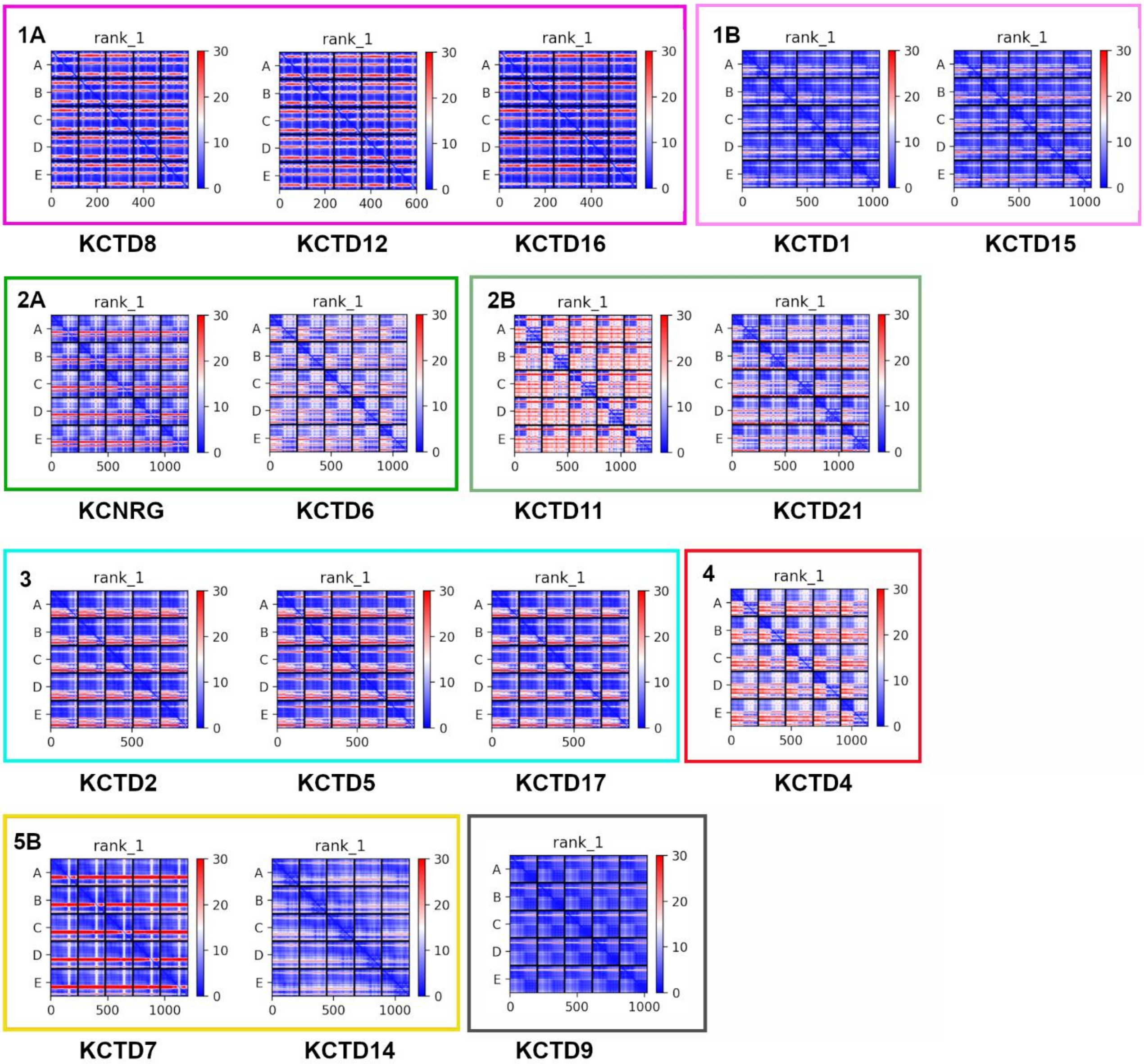


**Figure S3.** PAE matrices of all reliable pentamers of full-length KCTDs. For the sub-cluster 1A, due to the separation of a long disordered link between the two domains of the proteins, predictions have been conducted separately on the individual domains and here we report the matrices for the C-terminal domain pentamers only.

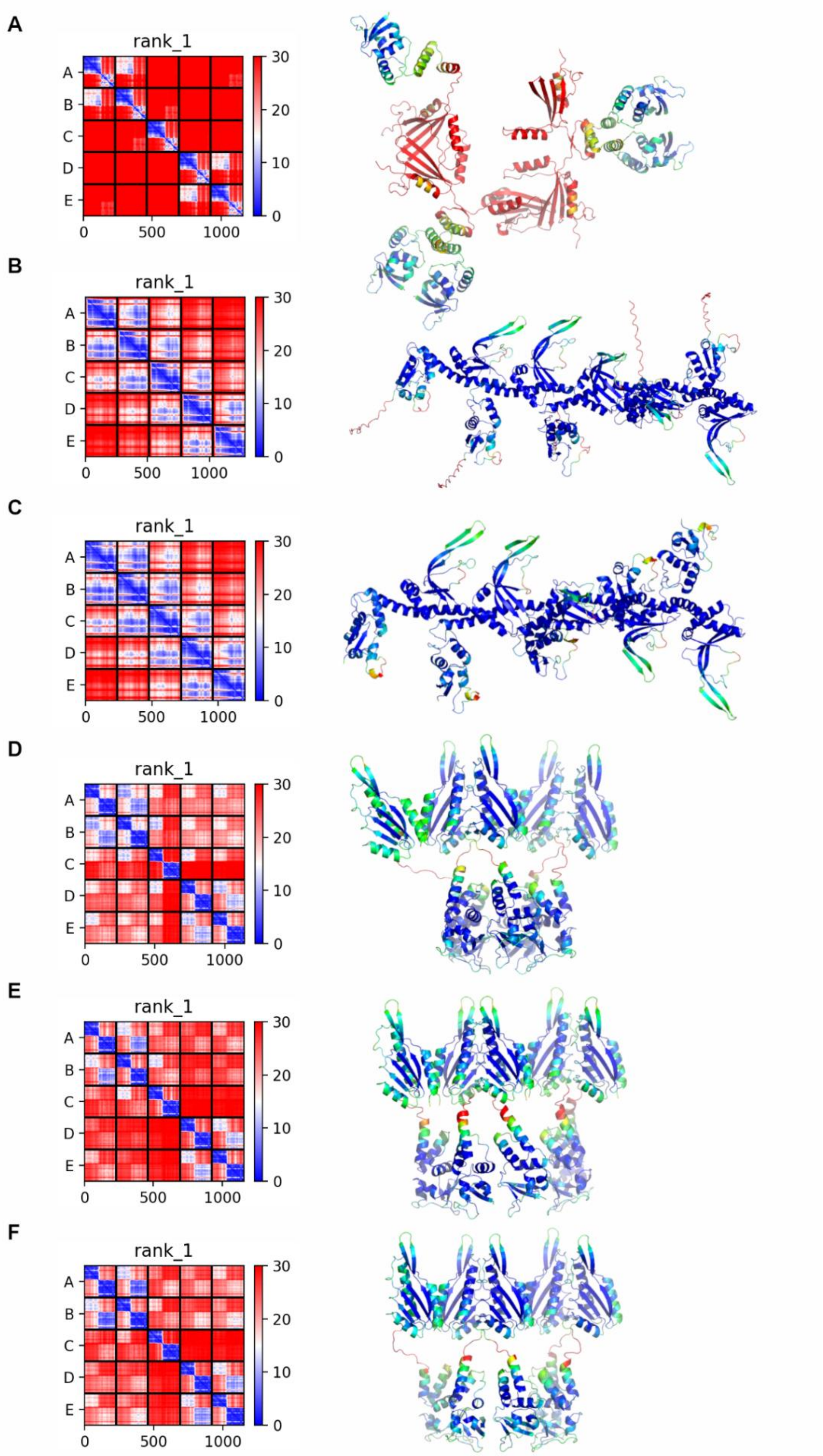


**Figure S4.** PAE matrices and unreliable structures of pentameric assemblies of full-length KCTDs: (**A**) KCTD18; (**B**) BTBD10; (**C**) KCTD20; (**D**) KCTD10; (**E**) KCTD13; (**F**) TNFAIP1.

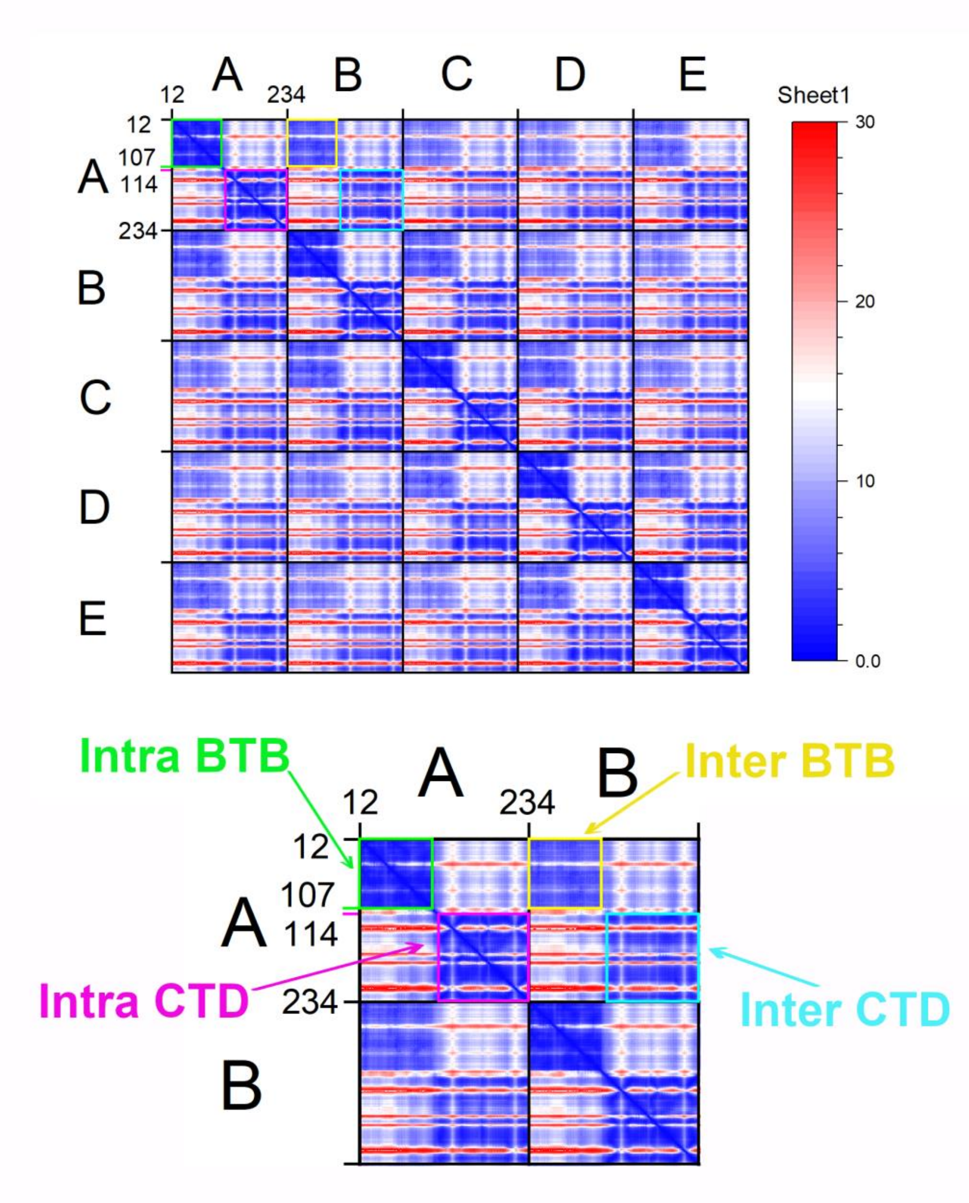


**Figure S5.** Quantification of the reliability of the predictions through the analyses of the matrices: KCTD6 as example. The BTB domain includes residues 12–107 whereas the CTD domain includes residues 114–234.

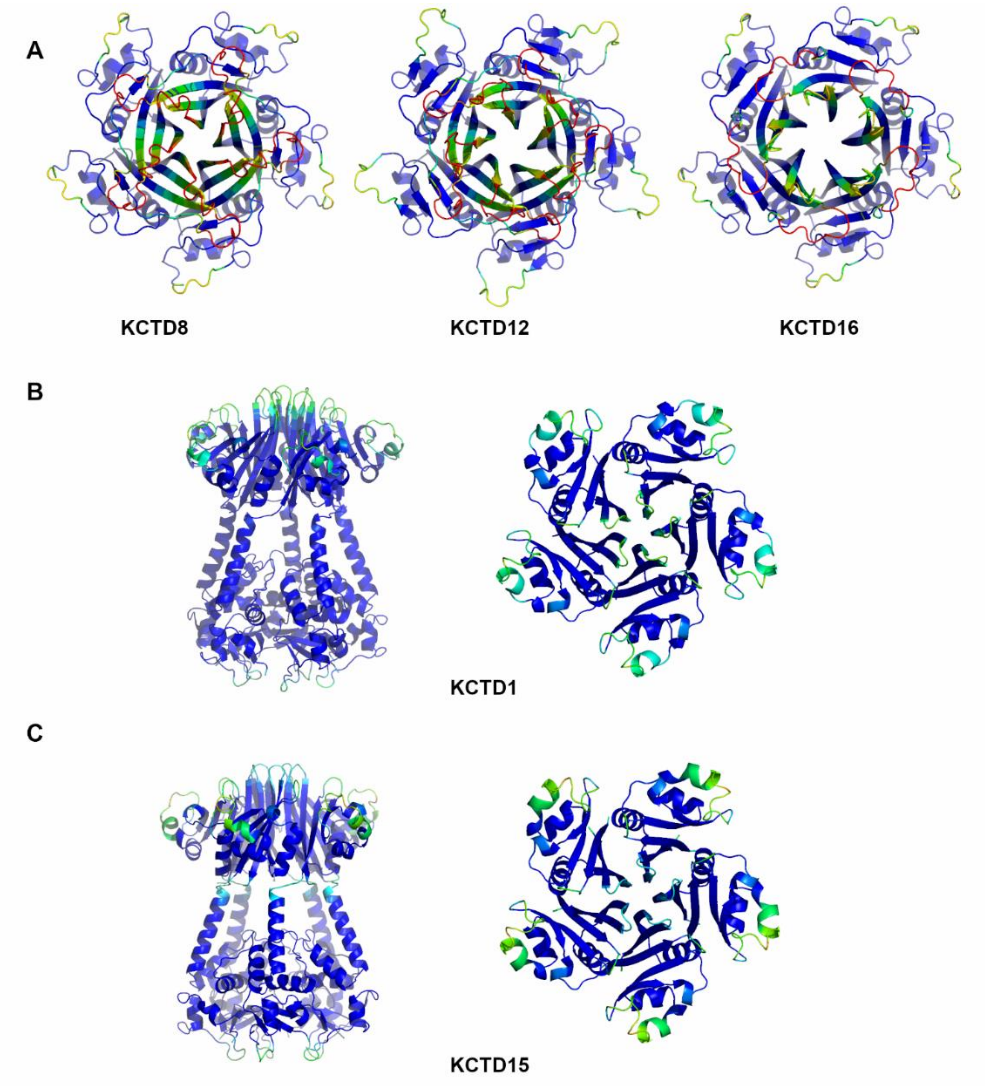


**Figure S6.** Schematic representation of AF structures of members of Cluster 1. (**A**) sub-cluster 1A; (**B**) KCTD1 full-length; (**C**) KCTD15 full-length. In panel A the CTD predicted models are shown (see main text). In panels B-C, two views (on the left panel the full-length pentameric assembly, on the right panel only the CTD domains) are shown. Structural models are colored following AF per-residue confidence metric (pLDDT).

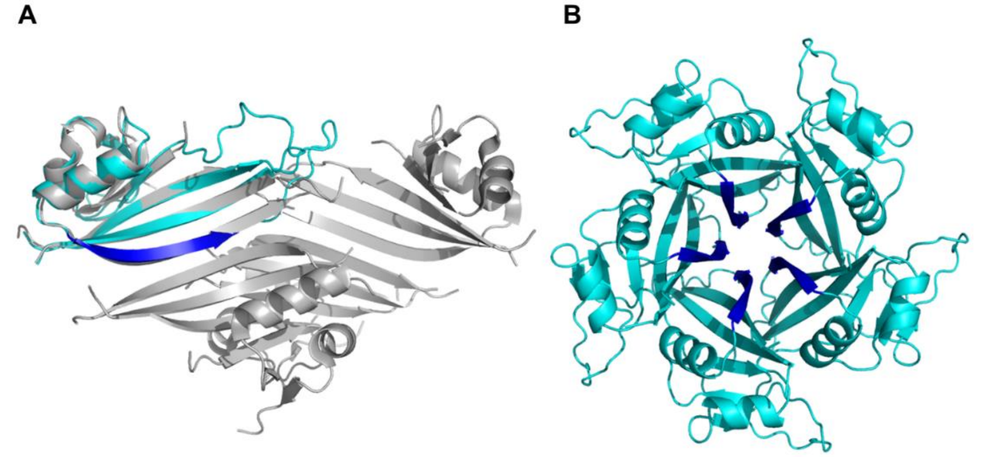


**Figure S7.** KCTD8 protein. (**A**) Experimental structure of the KCTD8 CTD domain in a tetrameric assembly (grey, PDB entry 6g57); one chain (cyan) of the AF pentameric CTD domain is

superimposed on a single experimental chain. (**B**) AF pentameric CTD assembly. The exposed β-strand in the channel is highlighted in blue.

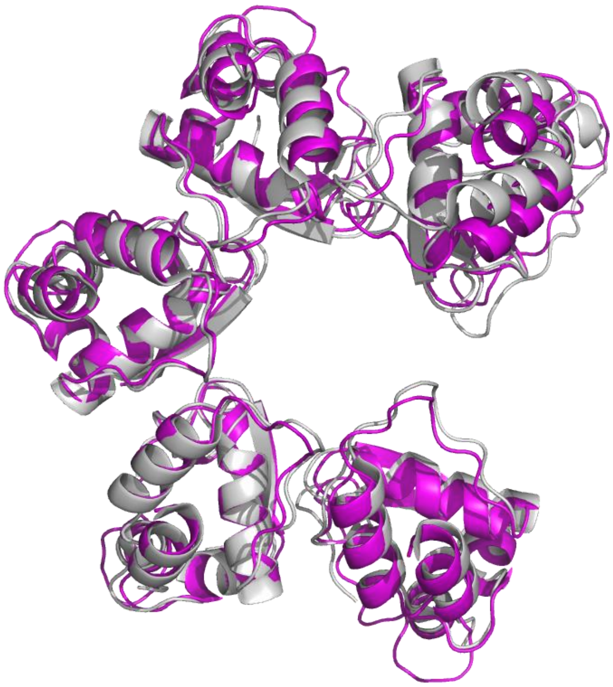

**Figure S8.** KCTD16 protein. Structural superimposition of the experimental structure (grey, PDB entry 6ocr) of the pentameric BTB assembly with the predicted open BTB assembly (magenta) extracted from the AF full-length model. The RMSD value computed on the $C^{\alpha}$ atoms is 1.25 Å (425 atoms aligned).

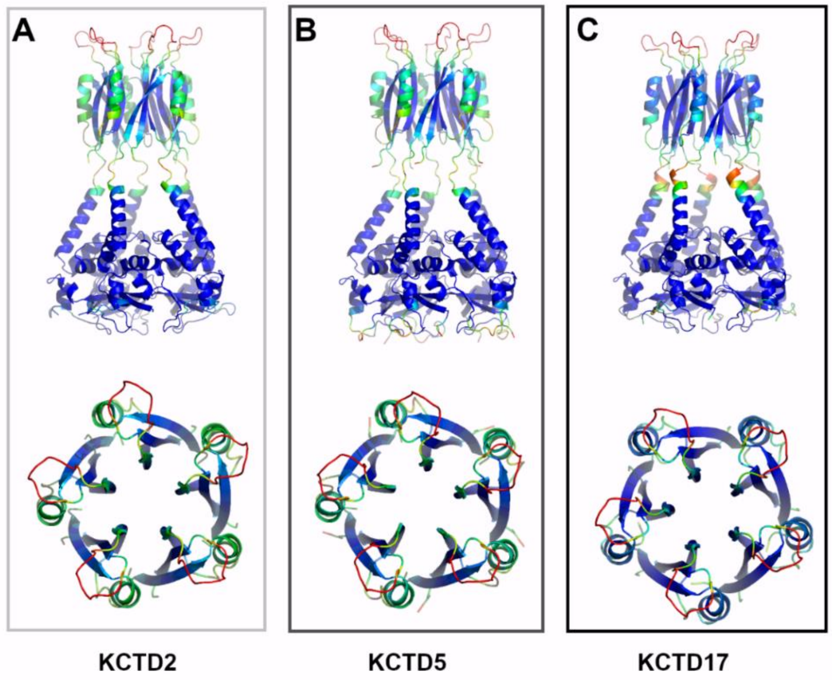


**Figure S9.** Schematic representation of AF structures of members of Cluster 3. (**A**) KCTD2; (**B**) KCTD5; (**C**) KCTD17. Two views (on the top the full-length pentameric assembly, on the bottom only the CTD domains) are shown. Structural models are colored following AF per-residue confidence metric (pLDDT).

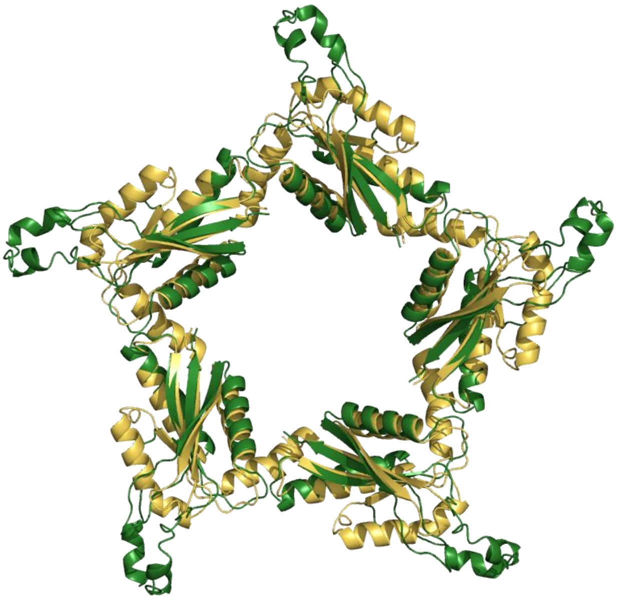

**Figure S10.** Structural superimposition of the CTDs of members of sub-cluster 5B: KCTD7 (dark green) and KCTD14 (gold). The RMSD value computed on the C$^{\alpha}$ atoms is 1.96 Å (355 atoms aligned).

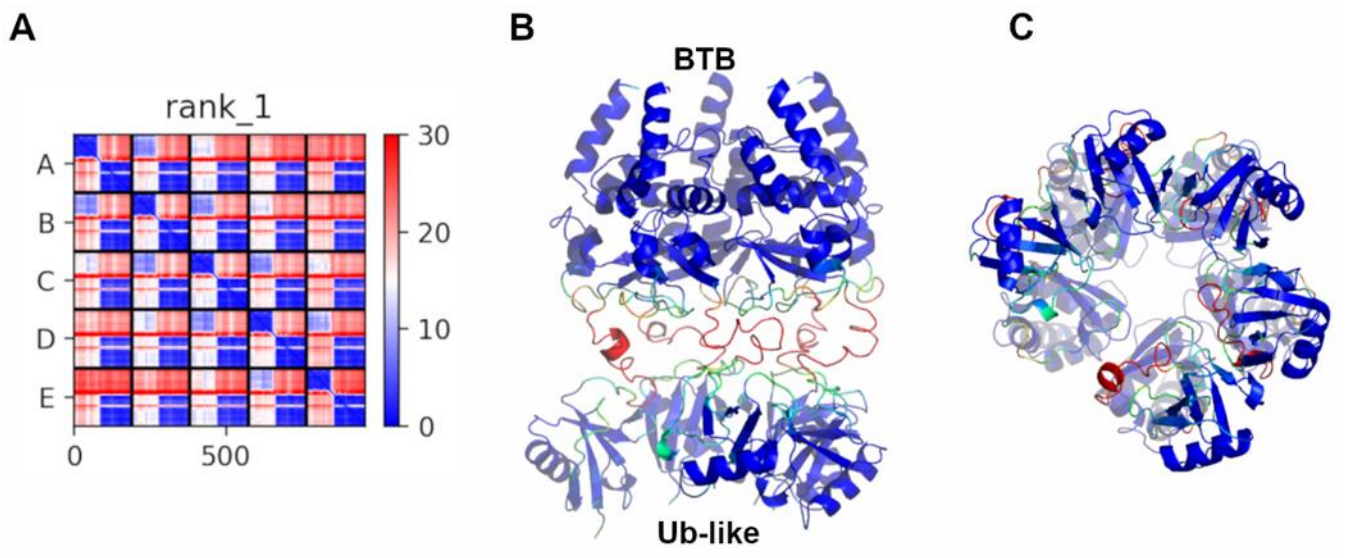


**Figure S11.** AF model of KCTD9 sequence including the N-terminal ubiquitin-like domain (residues 1–72) and the BTB domain (residues 89–191). (**A**) PAE matrix of the AF rank 1 predicted model; (**B**) Lateral view of the structural model (colored following AF per-residue confidence metric pLDDT); (C) Top view from the N-terminal Ub-like domain.

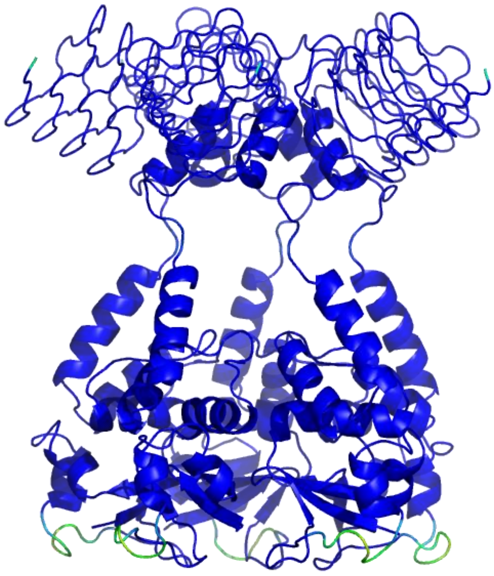

**Figure S12.** Schematic representation of the AF model of full-length KCTD9 with a reduced CTD domain (residues 89–282). The structural model is colored following AF per-residue confidence metric (pLDDT).

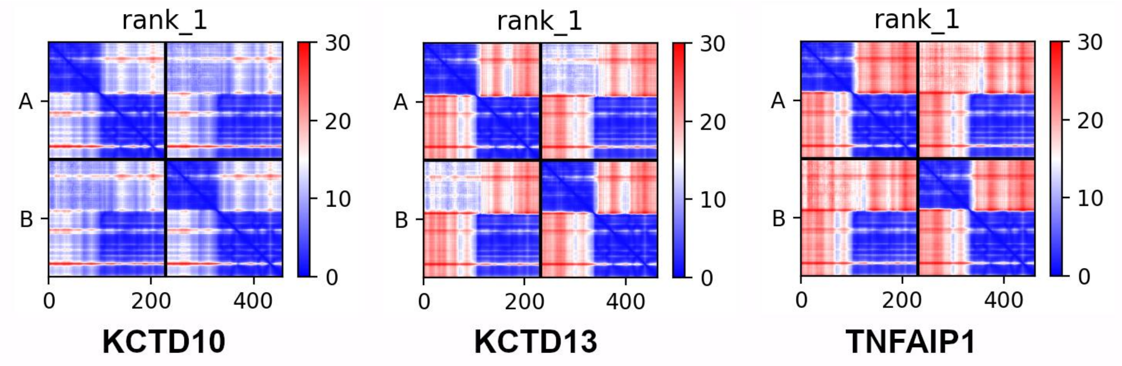


**Figure S13.** PAE matrices of Cluster 6 dimers (KCTD10, KCTD13 and TNFIAP1). Only the best AF prediction (rank 1) is shown.

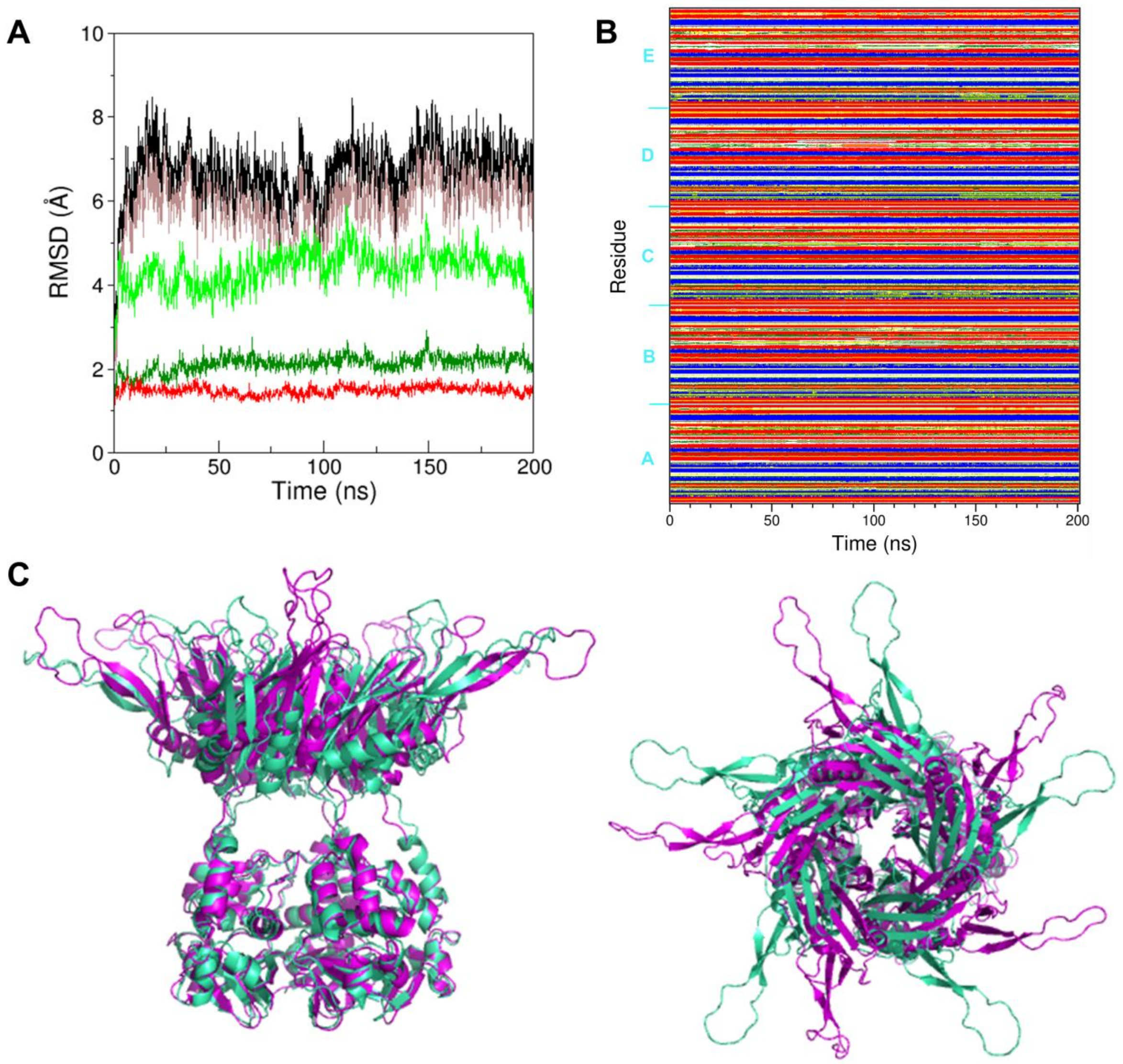


**Figure S14.** Structure stability of the pentameric AF model of KCNRG in the MD simulation. (**A**) RMSD values (on $C^{\alpha}$ atoms) of trajectory structures against the starting model. The RMSDs are calculated on the full-length structure (black) and on the single domains (BTB in red and CTD in green). RMSDs have also been computed by excluding the large loop regions of the CTD (full-length in light brown, CTD in dark green); (**B**) Time evolution of the secondary structure content (DSSP color code); (**C**) Structural alignments between the starting MD model (light green) and the frame (magenta) closest to the average structure computed on the equilibrated region of the trajectory. Two views are shown (on the left a lateral view, on the right a top view from the CTD).

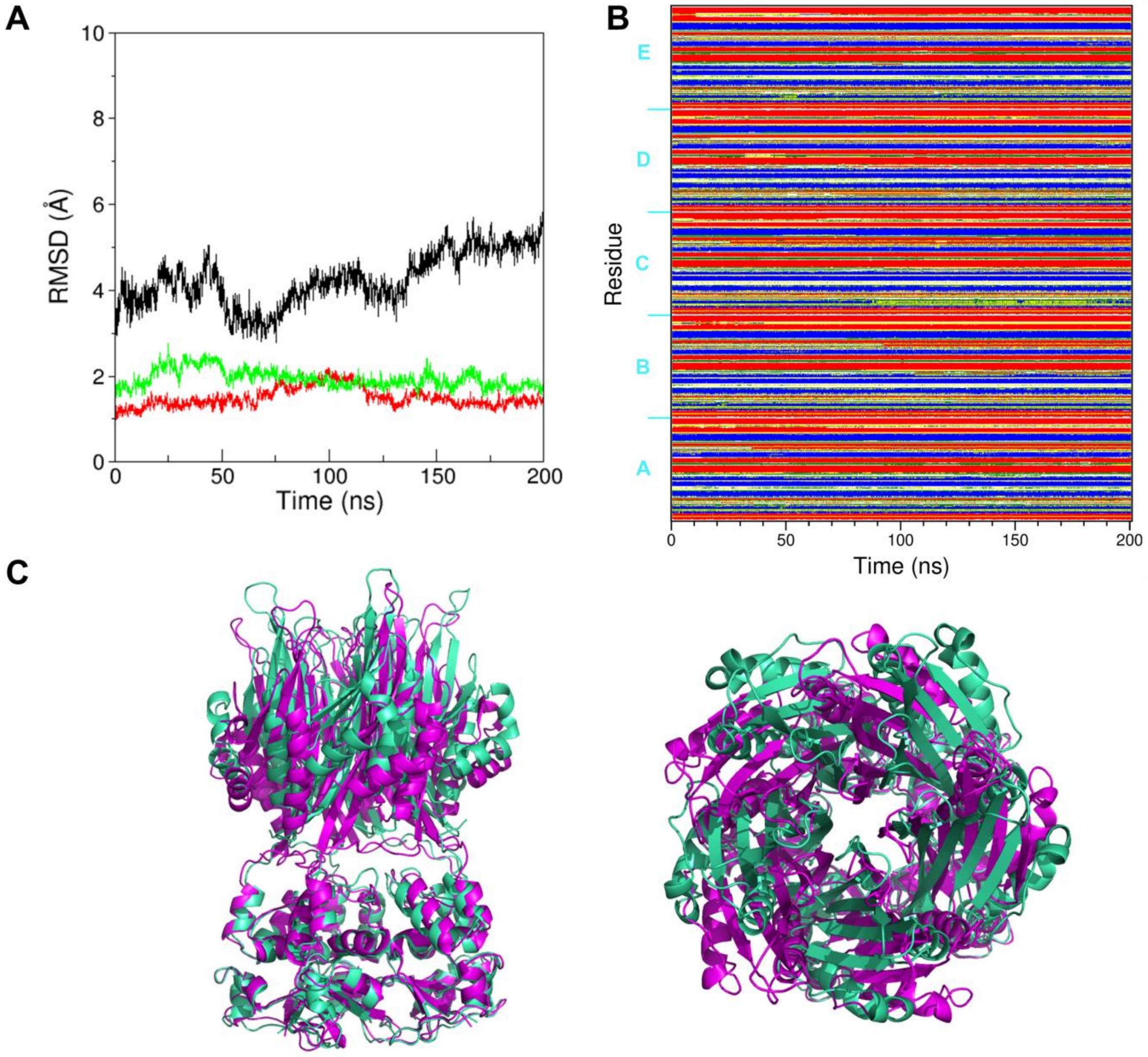


**Figure S15.** Structure stability of the pentameric AF model of KCTD6 in the MD simulation. (**A**) RMSD values (on $C^{\alpha}$ atoms) of trajectory structures against the starting model. The RMSDs are calculated on the full-length structure (black) and on the single domains (BTB in red and CTD in green); (**B**) Time evolution of the secondary structure content (DSSP color code); (**C**) Structural alignments between the starting MD model (light green) and the frame (magenta) closest to the average structure computed on the equilibrated region of the trajectory. Two views are shown (on the left a lateral view, on the right a top view from the CTD).

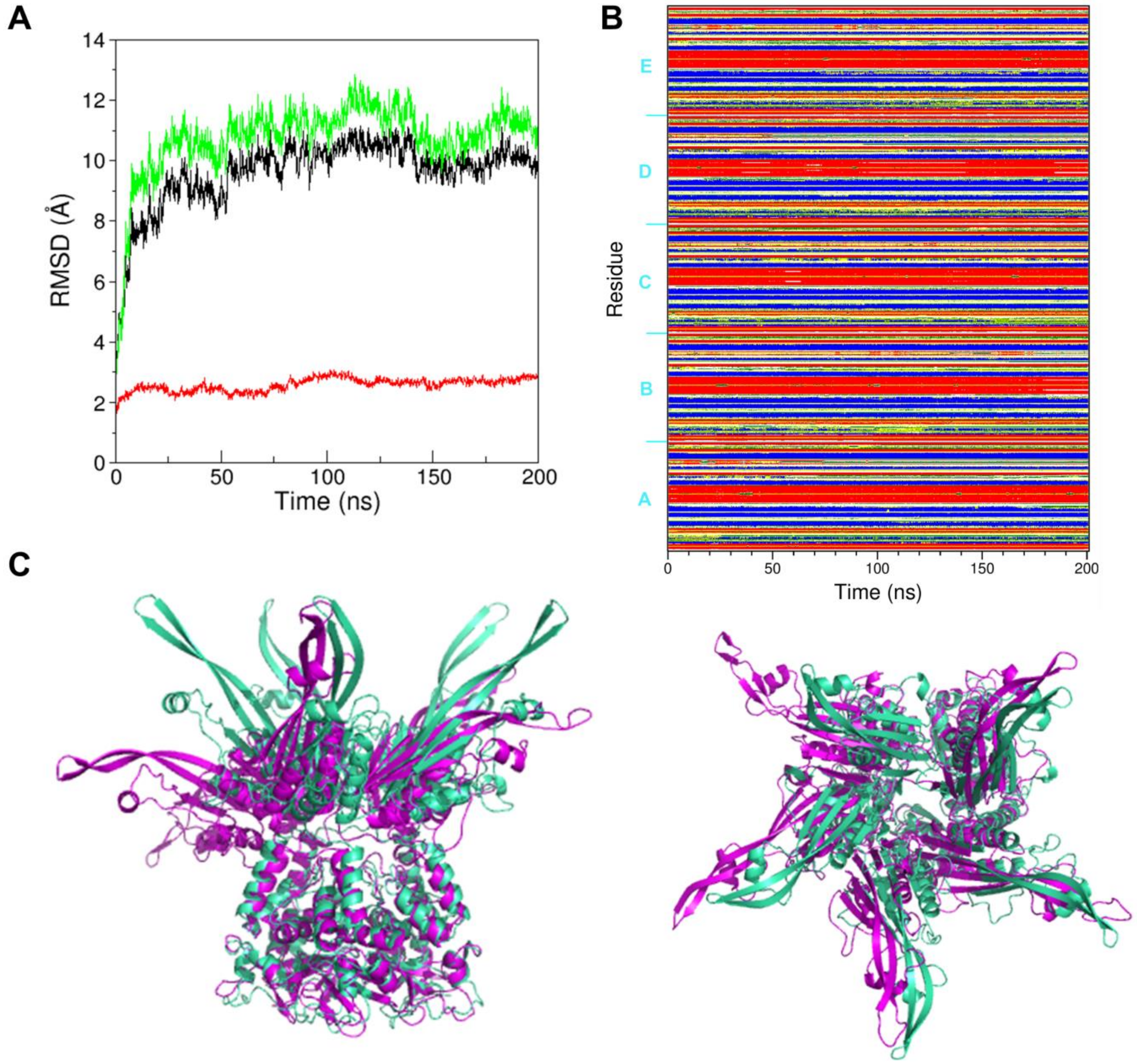


**Figure S16.** Structure stability of the pentameric AF model of KCTD11 in the MD simulation. (**A**) RMSD values (on C$^{\alpha}$ atoms) of trajectory structures against the starting model. The RMSDs are calculated on the full-length structure (black) and on the single domains (BTB in red and CTD in green); (**B**) Time evolution of the secondary structure content (DSSP color code); (**C**) Structural alignments between the starting MD model (light green) and the frame (magenta) closest to the average structure computed on the equilibrated region of the trajectory. Two views are shown (on the left a lateral view, on the right a top view from the CTD).

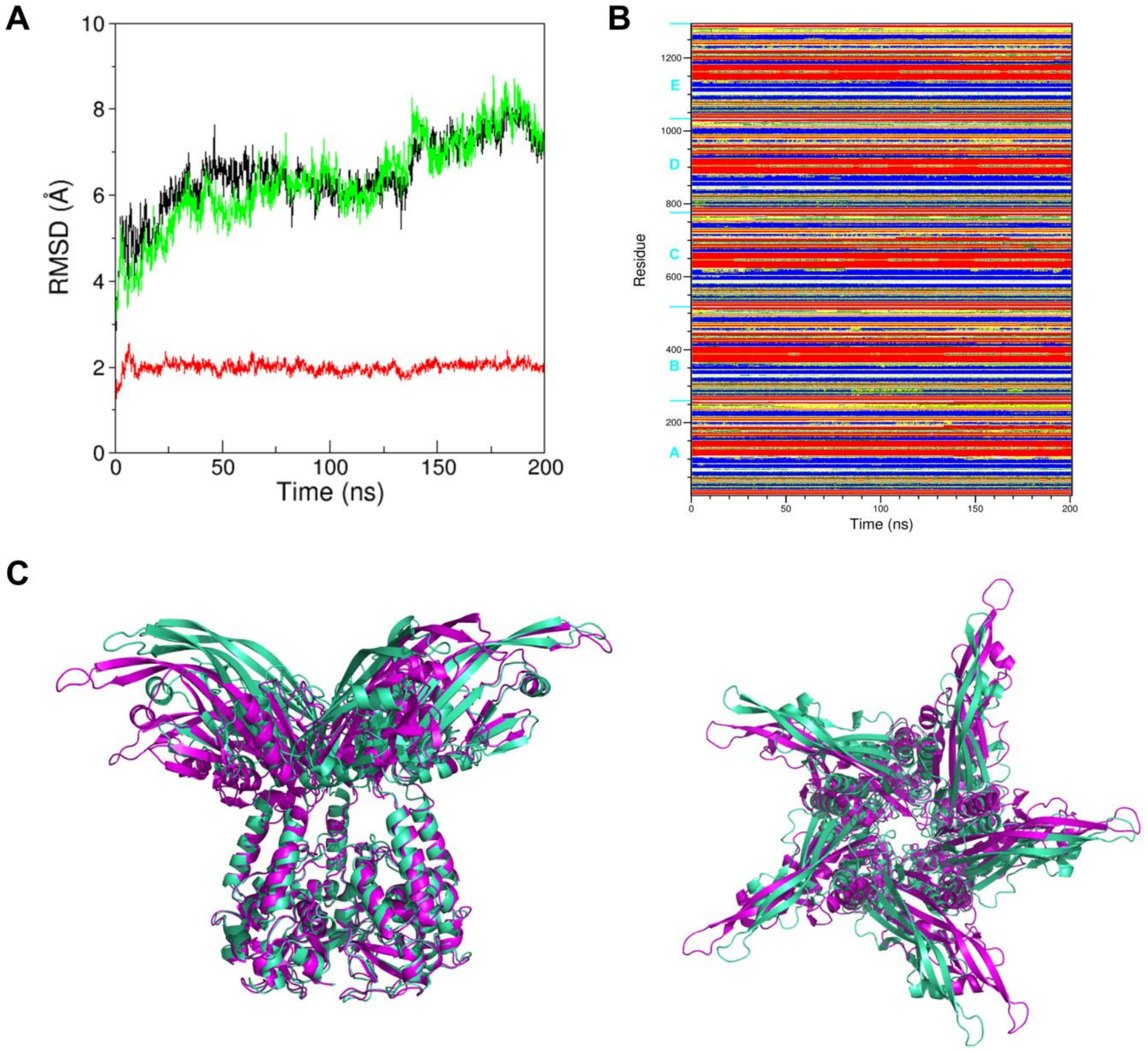


**Figure S17.** Structure stability of the pentameric AF model of KCTD21 in the MD simulation. (**A**) RMSD values (on $C^{\alpha}$ atoms) of trajectory structures against the starting model. The RMSDs are calculated on the full-length structure (black) and on the single domains (BTB in red and CTD in green); (**B**) Time evolution of the secondary structure content (DSSP color code); (**C**) Structural alignments between the starting MD model (light green) and the frame (magenta) closest to the average structure computed on the equilibrated region of the trajectory. Two views are shown (on the left a lateral view, on the right a top view from the CTD).

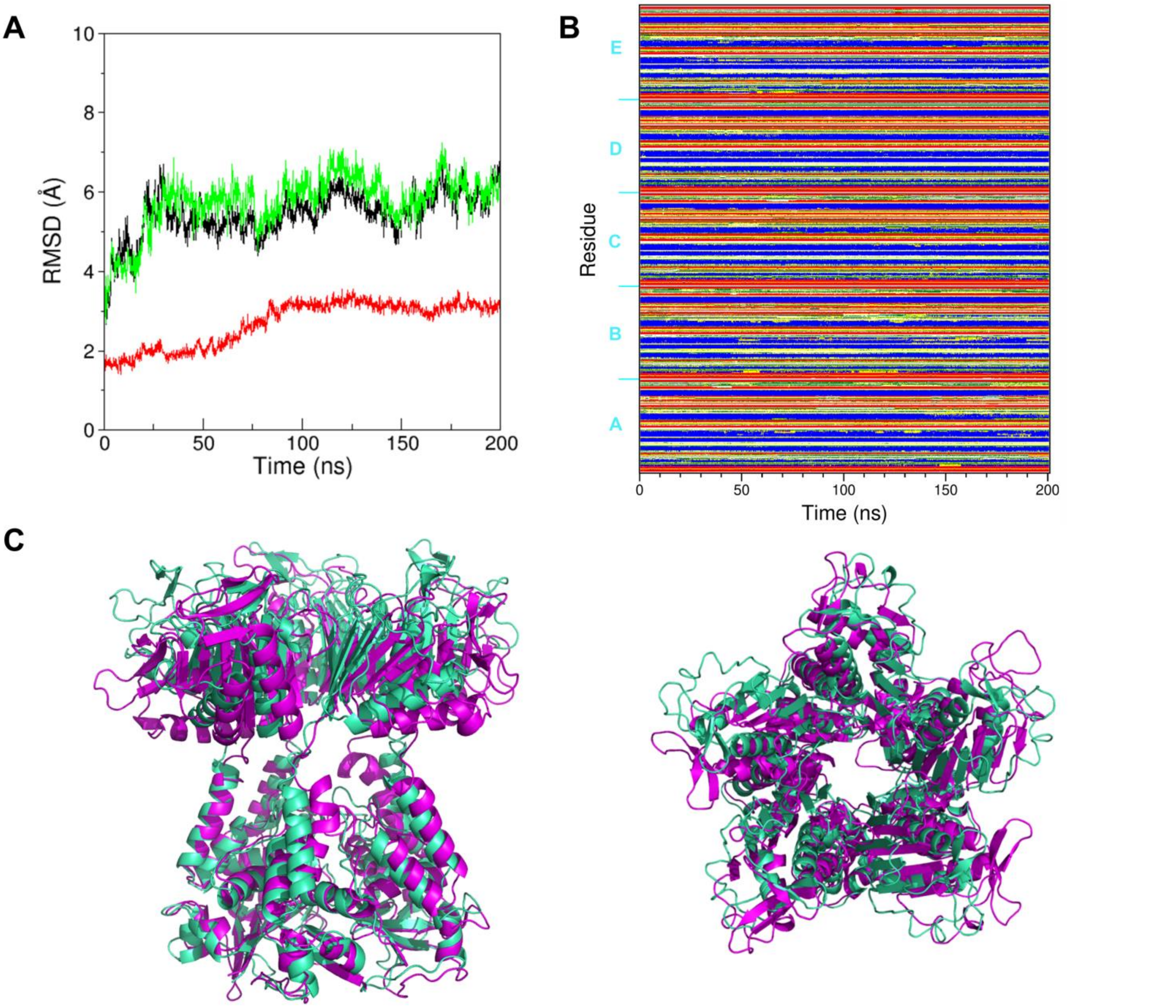


**Figure S18.** Structure stability of the pentameric AF model of KCTD4 in the MD simulation. (**A**) RMSD values (on $C^{\alpha}$ atoms) of trajectory structures against the starting model. The RMSDs are calculated on the full-length structure (black) and on the single domains (BTB in red and CTD in green); (**B**) Time evolution of the secondary structure content (DSSP color code); (**C**) Structural alignments between the starting MD model (light green) and the frame (magenta) closest to the average structure computed on the equilibrated region of the trajectory. Two views are shown (on the left a lateral view, on the right a top view from the CTD).

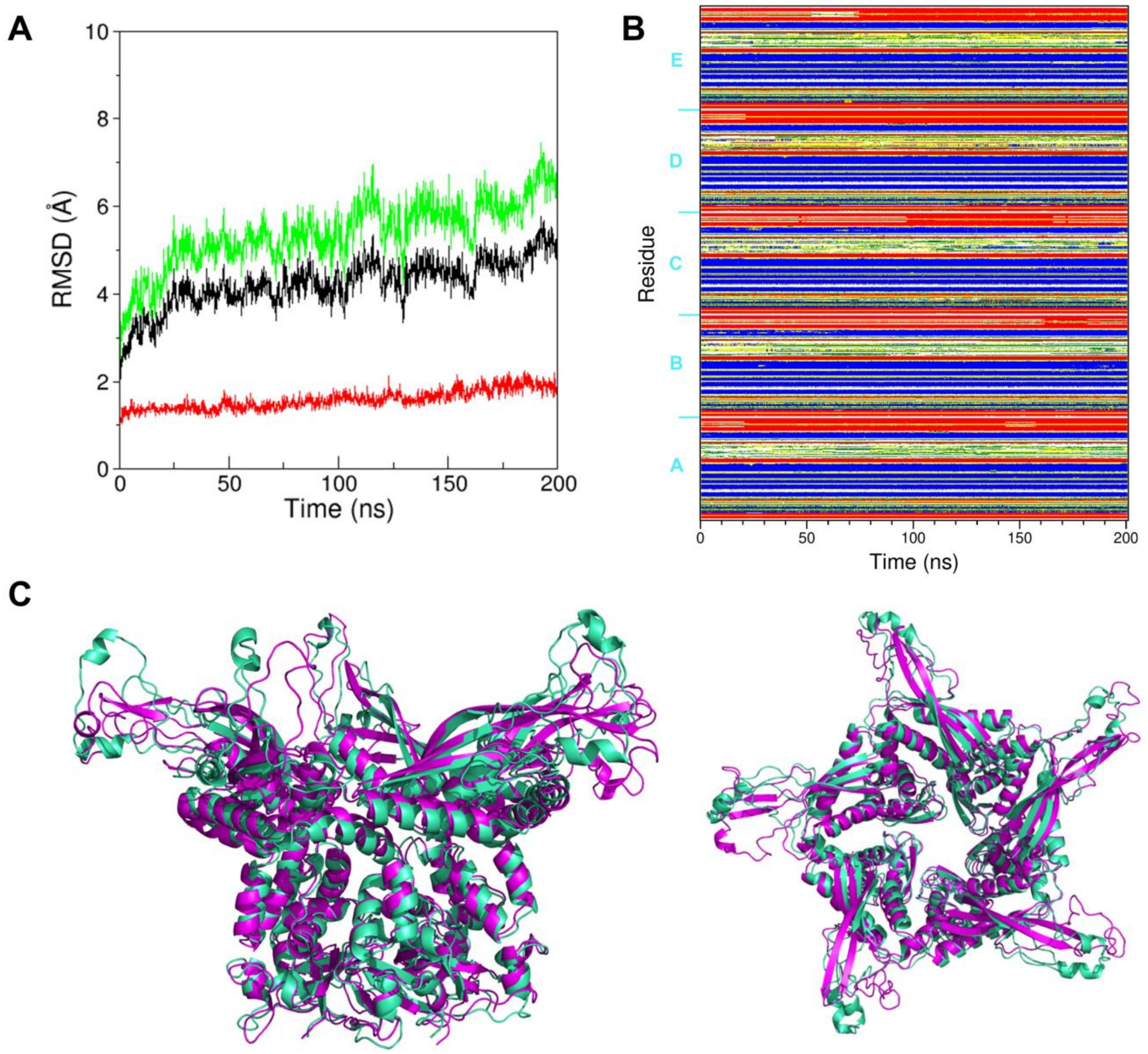


**Figure S19.** Structure stability of the pentameric AF model of KCTD7 in the MD simulation. (**A**) RMSD values (on $C^{\alpha}$ atoms) of trajectory structures against the starting model. The RMSDs are calculated on the full-length structure (black) and on the single domains (BTB in red and CTD in green); (**B**) Time evolution of the secondary structure content (DSSP color code); (**C**) Structural alignments between the starting MD model (light green) and the frame (magenta) closest to the average structure computed on the equilibrated region of the trajectory. Two views are shown (on the left a lateral view, on the right a top view from the CTD).

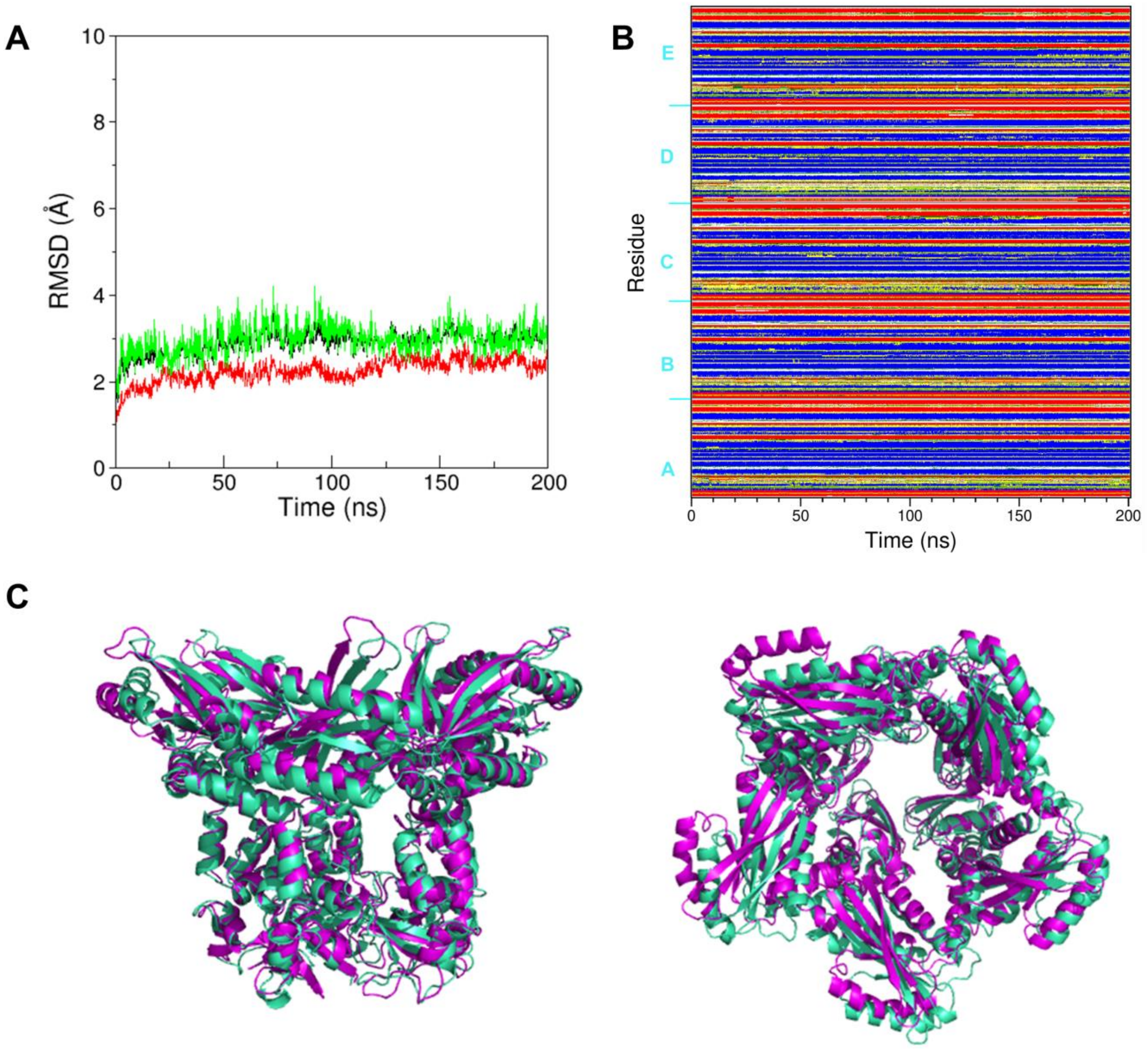


**Figure S20.** Structure stability of the pentameric AF model of KCTD14 in the MD simulation. (**A**) RMSD values (on $C^{\alpha}$ atoms) of trajectory structures against the starting model. The RMSDs are calculated on the full-length structure (black) and on the single domains (BTB in red and CTD in green); (**B**) Time evolution of the secondary structure content (DSSP color code); (**C**) Structural alignments between the starting MD model (light green) and the frame (magenta) closest to the average structure computed on the equilibrated region of the trajectory. Two views are shown (on the left a lateral view, on the right a top view from the CTD).

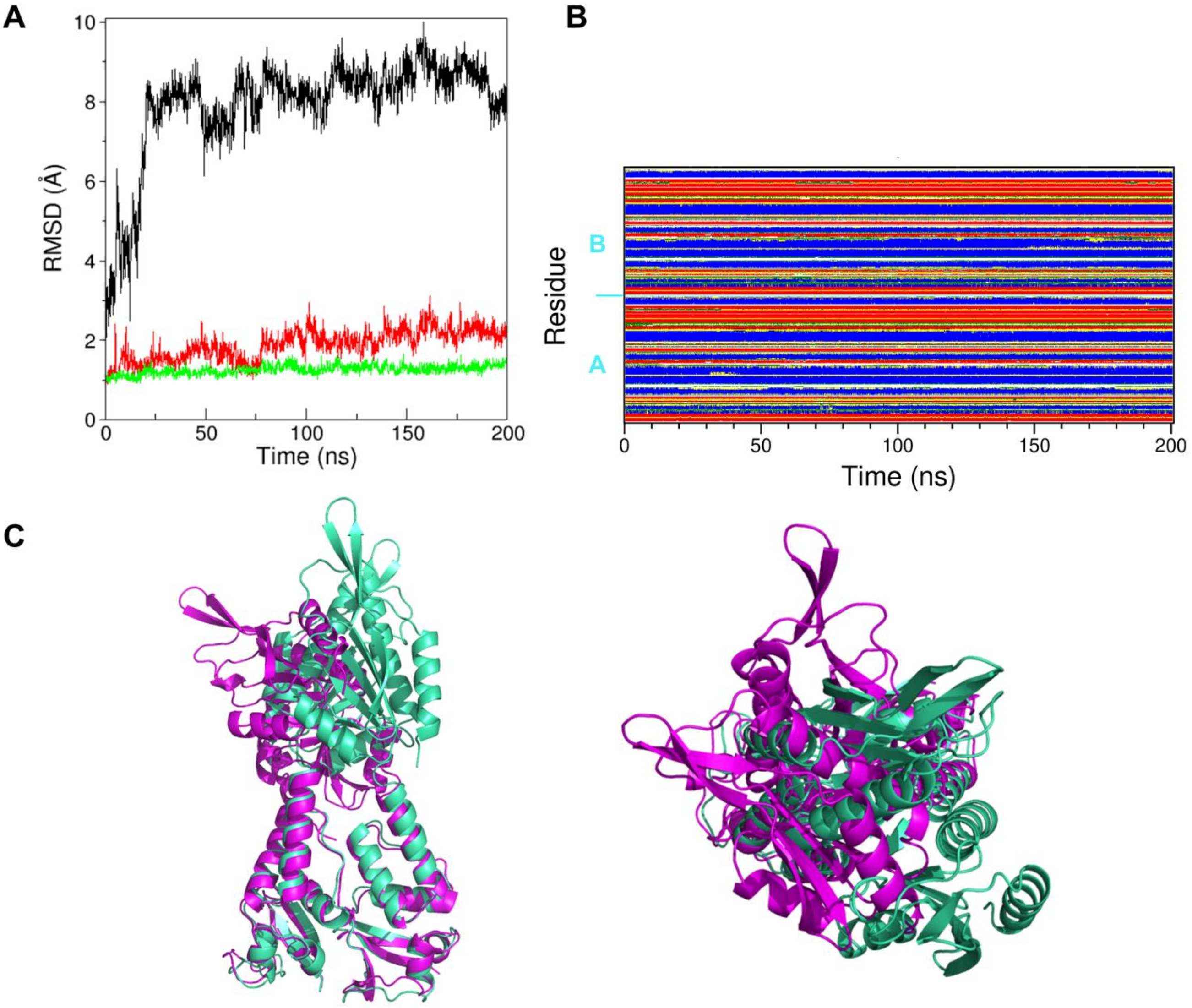


**Figure S21.** Structure stability of the dimeric AF model of KCTD13 in the MD simulation. (**A**) RMSD values (on $C^{\alpha}$ atoms) of trajectory structures against the starting model. The RMSDs are calculated on the full-length structure (black) and on the single domains (BTB in red and CTD in green); (**B**) Time evolution of the secondary structure content (DSSP color code); (**C**) Structural alignments between the starting MD model (light green) and the frame (magenta) closest to the average structure computed on the equilibrated region of the trajectory. Two views are shown (on the left a lateral view, on the right a top view from the CTD).
